# Absolute Humidity and the Seasonal Onset of Influenza in the Continental United States

**DOI:** 10.1371/journal.pbio.1000316

**Published:** 2010-02-23

**Authors:** Jeffrey Shaman, Virginia E. Pitzer, Cécile Viboud, Bryan T. Grenfell, Marc Lipsitch

**Affiliations:** 1 College of Oceanic and Atmospheric Sciences, Oregon State University, Corvallis, Oregon, United States of America; 2 Fogarty International Center, National Institutes of Health, Bethesda, Maryland, United States of America; 3 Center for Infectious Disease Dynamics, Pennsylvania State University, State College, Pennsylvania, United States of America; 4 Department of Ecology and Evolutionary Biology, Princeton University, Princeton, New Jersey, United States of America; 5 Woodrow Wilson School, Princeton University, Princeton, New Jersey, United States of America; 6 Center for Communicable Disease Dynamics, Harvard School of Public Health, Harvard University, Boston, Massachusetts, United States of America; 7 Department of Epidemiology, Harvard School of Public Health, Harvard University, Boston, Massachusetts, United States of America; 8 Department of Immunology and Infectious Diseases, Harvard School of Public Health, Harvard University, Boston, Massachusetts, United States of America; Imperial College London, United Kingdom

## Abstract

Much of the observed wintertime increase of mortality in temperate regions is attributed to seasonal influenza. A recent reanalysis of laboratory experiments indicates that absolute humidity strongly modulates the airborne survival and transmission of the influenza virus. Here, we extend these findings to the human population level, showing that the onset of increased wintertime influenza-related mortality in the United States is associated with anomalously low absolute humidity levels during the prior weeks. We then use an epidemiological model, in which observed absolute humidity conditions temper influenza transmission rates, to successfully simulate the seasonal cycle of observed influenza-related mortality. The model results indicate that direct modulation of influenza transmissibility by absolute humidity alone is sufficient to produce this observed seasonality. These findings provide epidemiological support for the hypothesis that absolute humidity drives seasonal variations of influenza transmission in temperate regions.

## Abstract 2

Here, the authors demonstrate that variations of absolute humidity explain both the onset of wintertime influenza transmission and the overarching seasonality of this pathogen in temperate regions.

## Author Summary

The origin of seasonality in influenza transmission is both of palpable public health importance and basic scientific interest. Here, we present statistical analyses and a mathematical model of epidemic influenza transmission that provide strong epidemiological evidence for the hypothesis that absolute humidity (AH) drives seasonal variations of influenza transmission in temperate regions. We show that the onset of individual wintertime influenza epidemics is associated with anomalously low AH conditions throughout the United States. In addition, we use AH to modulate the basic reproductive number of influenza within a mathematical model of influenza transmission and compare these simulations with observed excess pneumonia and influenza mortality. These simulations capture key details of the observed seasonal cycle of influenza throughout the US. The results indicate that AH affects both the seasonality of influenza incidence and the timing of individual wintertime influenza outbreaks in temperate regions. The association of anomalously low AH conditions with the onset of wintertime influenza outbreaks suggests that skillful, short-term probabilistic forecasts of epidemic influenza could be developed.

## Introduction

In temperate regions, wintertime influenza epidemics are responsible for considerable morbidity and mortality [1]. These seasonal epidemics are maintained by the gradual antigenic drift of surface antigens, which enables the influenza virus to evade host immune response [2]. Recent influenza epidemics have resulted from the cocirculation of three virus (sub)types, A/H1N1, A/H3N2, and B, with one generally predominant locally in a given winter [3]–[5]. In contrast, influenza pandemic activity can occur any time of year, including during spring or summer months, in the rare instances when a novel virus to which humans have little or no immunity jumps from avian or mammalian hosts into the human population, as in the on-going H1N1v pandemic [6]–[9]. Despite numerous reports describing wintertime transmission of epidemic influenza in temperate regions [10], our understanding of the mechanisms underlying influenza seasonal variation remains very limited.

**Figure 1:**
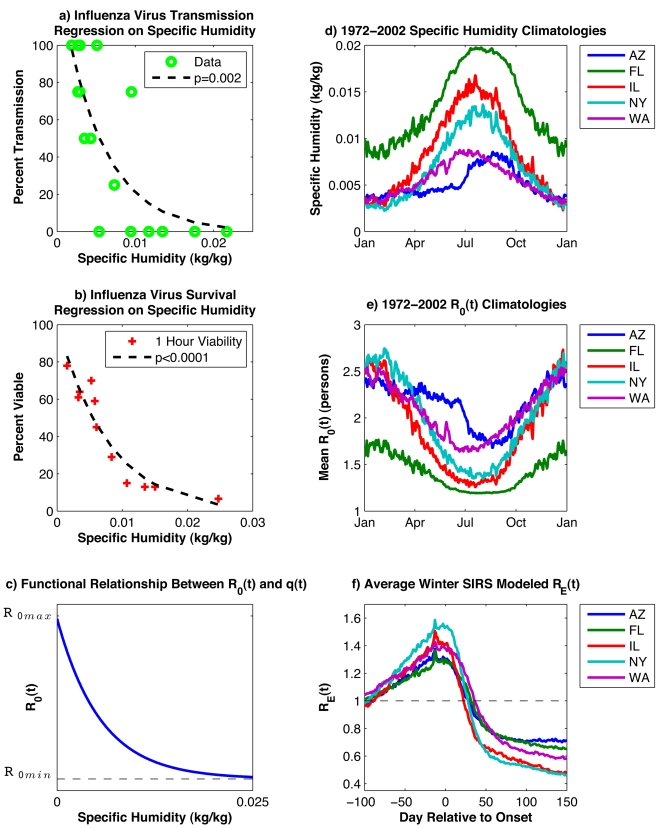
Analyses of laboratory data, environmental data, and SIRS model simulations. (A) Log-linear regression of guinea pig airborne influenza virus transmission data [14],[15] on specific humidity (a measure of AH); (B) log-linear regression of 1-h influenza virus survival data [28] on specific humidity; (C) functional relationship between *R*_0_(*t*) and *q*(*t*) per Equation 4; (D) 1972–2002 daily climatology of 2-m above-ground NCEP-NCAR reanalysis specific humidity [23] for Arizona, Florida, Illinois, New York state, and Washington state; (E) 1972–2002 average daily values of *R*_0_(*t*) derived from the specific humidity climatology using the best-fit parameter combination from SIRS simulations (*R*_0max_ = 3.52; *R*_0min_ = 1.12) and the functional form (Figure 1C and Equation 4); (F) average *R*_E_(*t*) for all wintertime outbreaks in the ten best-fit simulations at each state shown for 100 d prior to through 150 d post outbreak onset (minimum 400 infections/day during 2 wk prior; minimum 5,000 infections/day at least 1 d during subsequent 30 d). Figure 1A and 1B are redrawn from Shaman and Kohn [11] using specific humidity as the measure of AH.

Experimental studies suggest that influenza virus survival within aerosolized droplets is strongly associated with the absolute humidity (AH) of the ambient air, such that virus survival improves markedly as AH levels decrease [11]. A similar relationship is observed between AH and airborne influenza virus transmission among laboratory guinea pigs, in that transmission increases markedly as AH levels decrease (Figure 1). Within temperate regions of the world, AH conditions are minimal in winter and maximal in summer (Figure 1D). This seasonal cycle favors a wintertime increase of both influenza virus survival and transmission, and may explain the observed seasonal peak of influenza morbidity and mortality during winter. Annual wintertime mortality peaks are evident in the long-term mortality records of excess pneumonia and influenza (P&I) in the US, a robust indicator of the timing and impact of epidemics at national and local scales [4] (Figure S1).

Here, we develop epidemiological support for these previous laboratory-based findings implicating AH as a driver of seasonal influenza transmission. First, we analyze the spatial and temporal variation of epidemic influenza onset across the continental US, 1972–2002, and correlate this observed variability with records of AH for the same period and locations. Second, we show that a mathematical model of influenza transmission in the US can reproduce the spatial and temporal variation of epidemic influenza when daily AH conditions within each state are used to modulate the basic reproductive number, *R*_0_(*t*), of the influenza virus.

## Results

### AH and the Onset of Wintertime Influenza Outbreaks

Our first test of the hypothesis that low AH drives wintertime increases of influenza transmission is to assess whether the onset of the influenza epidemic each winter—which shows substantial annual variation (Figure S1)—corresponds to a period of unusually low AH. We define the onset of wintertime influenza as the date at which, for the 2 wk prior, the observed excess P&I mortality rate had been at or above a prescribed threshold level (e.g., 0.01 deaths/100,000 people/day). This onset date was identified separately for each of the 30 winters in the 1972–2002 observational record at each of the 48 contiguous states plus the District of Columbia (DC). We then examined the anomalous AH (*AH′*) conditions prior to and following these onset dates. *AH′* is the local daily deviation of AH from its 31-y mean for each day (as shown for five states in Figure 1D), defined as:
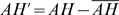
(1)where 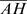 denotes the 1972–2002 daily average value. At temperate latitudes, such as in the US, wintertime AH levels are already much lower than summer (Figure 1D). By using *AH′*, we can determine whether the onset of wintertime influenza occurs when AH is above or below typical local daily AH levels.

**Figure 2:**
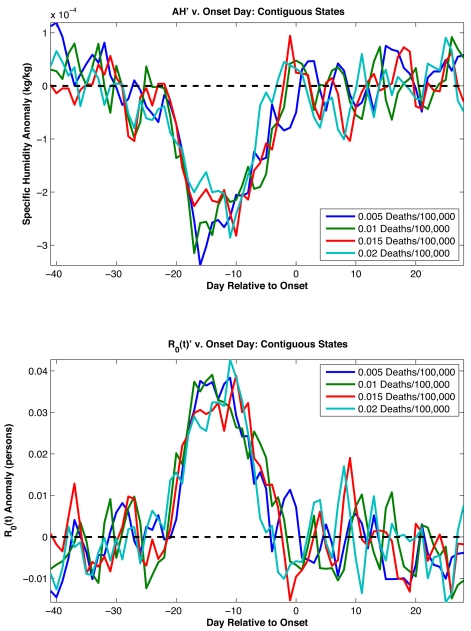
*AH′* associated with the observed onset of epidemic influenza. Top, plots of *AH′* averaged for the site-winters with an influenza outbreak showing the 6 wk prior to and 4 wk following outbreak onset. The conditions at each of the site-winters are defined based on the onset date for that site-winter. The onset dates are defined as the date at which wintertime observed excess P&I mortality had been at or above a prescribed threshold level for two continuous weeks (e.g., 0.01 deaths/100,000 people/day). Not every site-winter produced an outbreak as defined by a particular onset threshold. Depending on the threshold level used, 1,181–1,420 epidemics were identified among 1,470 possible (30 winters each for the 48 contiguous states plus the District of Columbia). Each solid line is the averaged *AH′* associated with influenza onset as defined by a different threshold mortality rate. The dashed line shows *AH′* = 0. Bottom, plot of *R*_0_(*t*) anomalies using the above *AH′* values. The *R*_0_(*t*) anomalies are calculated using the best combined-fit estimates of *R*_0max_ and *R*_0min_ (Table 1). The dashed line shows *R*_0_(*t*)**′** = 0.

Negative *AH′* values are typically observed beginning 4 wk prior to the onset of influenza epidemics (Figure 2), with the largest excursion occurring 17 d prior to onset. This result is robust to the choice of the mortality threshold level used to define onset date (from 0.001 to 0.02 excess P&I deaths/100,000 people/day). To assess the statistical significance of the association between negative wintertime *AH′* and epidemic onset, we bootstrapped the distribution of observed wintertime *AH′* records and found strong statistical support (*p*<0.0005, see Text S1). Depending on the threshold used to define onset, 55%–60% of onset dates demonstrate negative *AH′* averaged over the 4 wk prior to onset. Although highly statistically significant, this shift from the expected 50% likelihood is small. These findings indicate that negative *AH′* are not necessary for wintertime influenza onset but instead presage an increased likelihood of these onset events. In effect, negative *AH′* in the weeks prior to onset provide an additional increase of influenza virus survival and transmission over typical local wintertime levels and may further facilitate the spread of the virus.

Regional differences in the association of negative *AH′* with onset date are also evident. The association is strongest in the eastern US, in particular the Gulf region and the northeast (Figures S2, S3, and S4). Although the association does not reach statistical significance in much of the western US, *AH′* are typically negative during the weeks prior to onset in this region as well.

Next, we used the same approach to examine whether other potential environmental drivers of influenza are associated with wintertime influenza onset. The findings indicate that negative relative humidity (RH) and temperature anomalies, as well as positive solar insolation anomalies, are also associated with onset date (Table 1). However, the direction of the associations of the daily wintertime anomalies of solar insolation and RH with epidemic onset are contrary to the association between these environmental factors and epidemic activity at the seasonal time scale. Decreased solar insolation during the winter months is posited to increase influenza activity by decreasing host melatonin and vitamin D levels and thus host resistance [12],[13]; however, our findings indicate that influenza onset is associated with *increased* daily solar insolation anomalies. Similarly, RH is highest in winter [11], but influenza onset is associated with *low* RH anomalies.

**Table 1:** Association of daily anomalies in various environmental variables with wintertime influenza onset during 1972–2002 for the contiguous US.

Onset Threshold (Deaths/100,000/Day)	AH′ (1,000*kg/kg)	RH′ (%)	Temperature′ (Kelvin)	Solar Radiation′ (W/m^2^)
0.005	−0.138 (<0.00002)	−0.420 (0.00166)	−0.221 (0.00004)	0.431 (0.0397)
0.01	−0.124 (<0.00002)	−0.586 (0.00006)	−0.212 (0.00044)	0.547 (0.0068)
0.015	−0.114 (<0.00002)	−0.709 (<0.00002)	−0.178 (0.00398)	0.594 (0.0051)
0.02	−0.107 (<0.00002)	−0.639 (<0.00002)	−0.184 (0.00402)	0.316 (NS)

Four different onset thresholds are shown. Average values for each variable are for the period 4 to 0 wk prior to onset. Significance estimates based on bootstrapping are also shown in parentheses.

NS = not significant.

Specific weather patterns may explain the observed correlations between these meteorological anomalies and influenza onset. Anomalously low AH over the continental US is typically associated with excursions of colder air masses from the north. These air masses, which often follow a cold front, bring cloud-free skies (i.e., increased solar insolation) and reduced surface temperature and humidity levels. As the air mass moves southward, it slowly warms; however, unless it traverses a large open water source, AH does not increase substantially. As a consequence, anomalously low RH levels can develop within these air masses as well. Thus, the anomalies of solar insolation and RH could be noncausally linked with influenza outbreaks through their association with weather conditions that bring negative *AH′* to a region.

Temperature and AH are strongly correlated (Table S1); both are minimal in winter when influenza transmission is maximal and have negative anomalies associated with influenza onset, tendencies which agree with the associations determined from laboratory data [11],[14],[15]. To establish which of these variables is most critical for onset, we rely on previous laboratory analyses exploring the impact of both environmental factors that indicate AH is the essential determinant of influenza virus survival and transmission [11]. Furthermore, *AH′* is the only anomaly variable whose association with onset is significant at *p*<0.00002 for all four onset threshold levels (Table 1).

In addition, it should be noted that seasonal temperature conditions are often highly managed indoors, where most of the US population spends the bulk of its time. Average daily outdoor temperatures can differ over 20 °C from winter to summer, but seasonal heating and air conditioning greatly reduce this temperature cycle indoors. In contrast, AH possesses a large seasonal cycle both outdoors and indoors [11].

### Model Simulations of Influenza Seasonality

To further assess the hypothesis that AH is a fundamental driver of influenza seasonality, we examined whether a population-level model of influenza transmission forced by AH conditions could reproduce the observed seasonal patterns of P&I mortality. We simulated influenza transmission for five states representative of different climates within the US: Arizona, Florida, Illinois, New York, and Washington. The model considers three disease classes: susceptible, infected, recovered; to integrate the impact of waning immunity following antigenic drift, we allow individuals to go back to the susceptible class at a defined rate (SIRS model). Observed 1972–2002 daily AH conditions within each state are used to modulate the basic reproductive number, *R*_0_(*t*), of the influenza virus, i.e., the per generation transmission rate in a fully susceptible population. These daily fluctuations of *R*_0_(*t*) alter the transmission probability per contact within the SIRS model and thus affect influenza transmission dynamics. The SIRS model contains four free parameters: two (*R*_0max_ and *R*_0min_) that define the range of *R*_0_(*t*), one for the duration of immunity (*D*), and one for the duration of infectiousness (*L*).

If absolute humidity controls influenza seasonality, best-fit simulations with the AH-driven transmission model should meet the following criteria: 1) the mean annual model cycle of infection should match observations in each state; 2) these simulations should converge to similar parameter values, i.e., the virus response to AH should be consistent among states; and 3) AH modulation of transmission rates (*R*_0_(*t*)) within the model must match the large range implied by the laboratory data (Figure 1).

**Figure 3:**
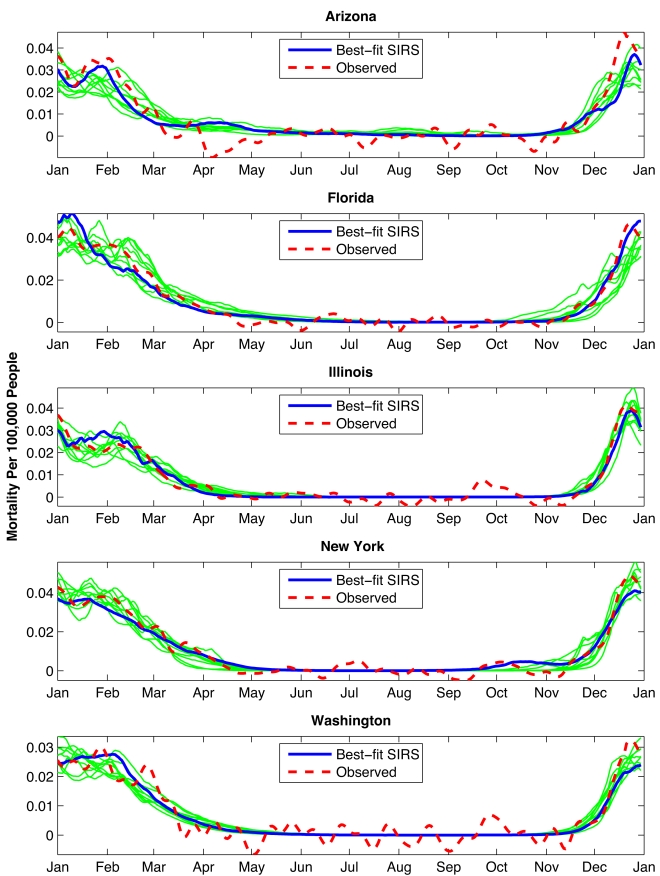
Mean annual cycles for the best-fit SIRS model simulations at the five state sites. Here, best-fit simulations were selected individually for each state based on RMS error after scaling the 31-y mean daily infection number to the 31-y mean observed daily excess P&I mortality rate. Thick blue line shows the best-fit simulation; thinner green lines show the next nine best simulations.

Multiple 31-y (1972–2002) simulations were run at each of the five states with randomly chosen parameter combinations. We then compared the mean annual cycle of daily infection from each simulation with a similar average of 1972–2002 observed excess P&I mortality rates [3],[4]. Best-fit model simulations at each site capture the observed seasonal cycle of influenza (Figure 3). These simulations produce not only the late-year rise in transmission and infection, but also the wintertime peak during early January, typically followed by a secondary peak during late February/early March. In both models and observations, the dual winter peaks are not typically seen in individual years; rather these epidemic trajectories reflect the averaging of individual wintertime outbreaks that peak anytime between December and April (Figure S5).

We also searched for the best-fit parameter combinations for all five sites evaluated together. The parameter combinations of these best “combined fits” are characterized by high *R*_0max_ (generally>2.8), high *R*_0min_ (>1), and low mean infectious period (2<*D*<4.2 d) (Figure S6; Table 2). Best-fit simulations at each of the five sites individually occupy a similar parameter space (Figures S7, S8, S9, S10, and S11; Table S2). In particular, these simulations converge to high *R*_0max_, which indicates a similar response to AH variability (see Text S1).

**Table 2:** Parameter combinations for the ten best-fit simulations at the Arizona, Florida, Illinois New York, and Washington state sites.

Rank	RMS Error	Correlation Coefficient (*r*)	*L* (Years)	*D* (Days)	*R*_0max_ (Persons/Person)	*R*_0min_ (Persons/Person)	Scaling Factor (×1e−4)
1	0.0070	0.85	5.35	3.24	3.52	1.12	1.70
2	0.0070	0.85	5.40	2.41	2.89	1.16	1.92
3	0.0075	0.83	3.28	4.18	3.40	1.22	1.04
4	0.0075	0.82	3.70	2.03	2.05	1.15	1.85
5	0.0075	0.82	7.77	2.59	3.69	1.30	2.28
6	0.0076	0.82	6.23	2.37	2.71	1.23	2.28
7	0.0076	0.82	6.05	2.56	3.79	1.06	1.83
8	0.0076	0.82	4.61	2.71	2.61	1.29	1.70
9	0.0076	0.81	7.39	2.85	3.69	1.27	2.22
10	0.0076	0.81	3.58	3.61	3.19	1.20	1.18

Five thousand simulations were performed at each site with the parameters *R*_0max_, *R*_0min_, *D*, and *L* randomly chosen from within specified ranges. Best-fit simulations were selected for the five sites in aggregate based on RMS error after scaling the 31-y mean daily infection number to the 31-y mean observed daily excess P&I mortality rate at each site. The scaling factor itself, representing mortality per infection, is also shown.

There is some correlation among SIRS model parameter values in simulations that fit the observed excess P&I mortality well. For instance, among better-fit simulations, *L* and *D* tend to be inversely related (Figures S6, S7, S8, S9, S10, and S11). In addition, broad regions of parameter space appear capable of producing high-quality, low root mean square (RMS) error simulations (Figure S6). The stochastic components of the SIRS model may contribute in part to this behavior. The flat goodness-of-fit within model parameter space indicates that no one parameter combination is strictly “best,” rather, a range of parameter combinations may produce good simulations of influenza transmission. These parameter ranges are: *L* = 3–8 y, *D* = 2–3.75 d, *R*_0max_ = 2.6–4, and *R*_0min_ = 1.05–1.30. We reran the SIRS model repeatedly sampling this approximate subset range of parameter space. Best-fit simulations from this subset range of parameter space (Table S3) were of similar quality and exhibited the same flat goodness-of-fit within model parameter space as the best-fit simulations presented in Table 2.

Because the SIRS model simulates only influenza-related infections, not deaths, a scaling factor is needed to compare model-simulated rates of infection with the observed excess P&I mortality rates. This scaling factor can be understood as the case fatality ratio, i.e., the probability of mortality given infection. Reassuringly, all best-fit simulations produce a scaling factor of the same order of magnitude and roughly consistent with the expected value of the case fatality ratio for P&I-related deaths (see Text S1).

The model also explains regional variations in influenza dynamics. Due to the modeled nonlinear relationship between *R*_0_(*t*) and AH (Figure 1C), the seasonal cycle of *R*_0_(*t*) is sensitive to both AH seasonal cycle amplitude and mean AH levels (Figure 1D and 1E). In Florida, mean AH levels are higher than for the other four states, but the seasonal AH cycle remains large and produces a seasonal *R*_0_(*t*) cycle of sufficient amplitude to generate an effective reproductive number, *R*_E_(*t*) = *R*_0_(*t*) *S(*t*)/*N*, greater than 1 (Figure 1F) and organize influenza epidemics preferentially during winter. Outbreak dynamics reinforce this phase organization in that wintertime epidemics confer immunity to a large proportion of the model population, which then reduces population-level susceptibility during the following summer when *R*_0_(*t*) is low. In Arizona and Washington state, the seasonal AH cycle is less than for the other three states, but average AH levels are lower, at a range where laboratory findings indicate sensitivity to variation in AH is greater; consequently *R*_0_(*t*) retains a sizeable seasonal cycle (Figure 1E). For all five states, the AH-driven seasonal variation of *R*_0_(*t*) is large enough that *R*_E_(*t*) is strongly modulated by AH conditions and exceeds 1 during winter as outbreaks develop (Figure 1F).

The humidity-driven SIRS simulations satisfy our three criteria for supporting the hypothesis that AH controls influenza seasonality in temperate regions. The simulations produce a consistent response in the five climatologically diverse US states using similar parameter values. The large sensitivity of simulated influenza transmission to AH is consistent with the analysis of laboratory experiments that show large changes in influenza virus survival and transmission in response to AH variability (Figure 1A and 1B).

### Cross-Validation of the Model Findings

To further validate the SIRS model findings, we determined whether the best-fit simulations derived from the five selected states could reproduce the seasonal cycles of influenza elsewhere in the US. The ten best combined-fit parameter combinations (Table 2) were used to perform 31-y (1972–2002) SIRS simulations at each of the contiguous 48 states plus DC.

The results of this cross-validation demonstrate good simulations of observed excess P&I mortality for a majority of states (average *r*>0.7, minimum *r*>0.5, see Methods and Table 3). Some states, particularly the sparsely populated western states perform less well. These states often have low workflow [4], which may reduce the rate of introduction of the virus each winter. In addition, heterogeneous AH fields across some states (particularly large ones) create some error due to simulation with a single average statewide AH value. Thirteen states in the continental US, including Arizona, possess low workflow rates [4]. Six of these 13 states are among the ten worst cross-validation performers; such a clustering is unlikely to occur by chance alone (*p*<0.005). In addition, seven of the ten worst performers are states with the ten lowest population densities (*p*<0.0001).

**Table 3:** Correlation coefficients for the contiguous US and District of Columbia of SIRS-simulated 1972–2002 influenza incidence with 1972–2002 observed excess P&I mortality.

	Ten-Run	Table 1 Ranked Parameter Combination									
State	Average	1	2	3	4	5	6	7	8	9	10
MO	0.92	0.93	0.95	0.87	0.89	0.91	0.93	0.85	0.93	0.95	0.97
OK^a^	0.91	0.94	0.95	0.95	0.96	0.84	0.90	0.88	0.85	0.91	0.93
KS	0.90	0.96	0.89	0.84	0.85	0.89	0.92	0.89	0.92	0.91	0.91
KY	0.89	0.91	0.88	0.87	0.90	0.83	0.88	0.88	0.95	0.87	0.92
AR	0.88	0.88	0.88	0.86	0.90	0.91	0.89	0.89	0.84	0.93	0.86
IA	0.88	0.91	0.88	0.92	0.81	0.91	0.91	0.86	0.85	0.89	0.87
PA	0.88	0.88	0.91	0.85	0.82	0.90	0.96	0.93	0.89	0.80	0.87
NH^a^	0.88	0.90	0.85	0.88	0.85	0.80	0.95	0.91	0.85	0.89	0.93
NY	0.88	0.88	0.82	0.92	0.78	0.87	0.85	0.94	0.95	0.96	0.81
IL	0.87	0.95	0.92	0.87	0.95	0.73	0.80	0.95	0.94	0.76	0.79
MA	0.86	0.89	0.86	0.87	0.93	0.69	0.77	0.90	0.87	0.90	0.97
IN	0.86	0.95	0.90	0.78	0.95	0.84	0.80	0.82	0.81	0.92	0.86
VA	0.86	0.80	0.75	0.83	0.81	0.93	0.84	0.93	0.79	0.94	0.94
NC	0.85	0.75	0.86	0.85	0.90	0.85	0.81	0.88	0.95	0.74	0.96
AL	0.85	0.83	0.84	0.85	0.97	0.69	0.90	0.89	0.97	0.76	0.85
MI	0.85	0.74	0.90	0.90	0.76	0.89	0.89	0.72	0.89	0.92	0.89
WV	0.84	0.83	0.84	0.90	0.86	0.92	0.81	0.84	0.79	0.86	0.75
WI	0.84	0.86	0.73	0.86	0.85	0.70	0.89	0.78	0.89	0.92	0.91
**NE**	0.84	0.91	0.78	0.78	0.87	0.94	0.87	0.67	0.81	0.93	0.81
ME^a^	0.84	0.72	0.91	0.76	0.86	0.86	0.92	0.79	0.81	0.89	0.86
TN	0.84	0.88	0.83	0.88	0.78	0.72	0.81	0.85	0.92	0.86	0.83
AZ^a^	0.82	0.69	0.80	0.85	0.82	0.82	0.90	0.84	0.86	0.81	0.84
CO^a^	0.82	0.85	0.90	0.80	0.84	0.60	0.86	0.72	0.86	0.92	0.91
MS	0.82	0.76	0.82	0.78	0.60	0.97	0.78	0.83	0.92	0.88	0.89
OH	0.82	0.76	0.87	0.91	0.79	0.79	0.76	0.66	0.80	0.91	0.91
TX	0.81	0.66	0.78	0.87	0.92	0.78	0.75	0.87	0.81	0.86	0.79
**SD** a	0.81	0.87	0.84	0.69	0.83	0.87	0.83	0.66	0.79	0.84	0.84
MN	0.81	0.84	0.72	0.74	0.81	0.83	0.85	0.80	0.76	0.86	0.84
MD	0.80	0.74	0.80	0.84	0.85	0.79	0.81	0.84	0.76	0.81	0.80
SC	0.80	0.80	0.84	0.89	0.84	0.76	0.92	0.55	0.84	0.86	0.74
WA	0.78	0.91	0.81	0.60	0.86	0.85	0.84	0.74	0.67	0.79	0.73
VT	0.77	0.68	0.84	0.87	0.76	0.76	0.87	0.72	0.73	0.64	0.82
GA	0.76	0.69	0.80	0.81	0.80	0.86	0.87	0.68	0.59	0.76	0.79
CT	0.76	0.75	0.75	0.83	0.76	0.71	0.66	0.87	0.80	0.65	0.83
LA	0.74	0.45	0.72	0.75	0.58	0.82	0.89	0.76	0.85	0.79	0.73
**NM** a	0.73	0.77	0.89	0.53	0.73	0.71	0.77	0.77	0.82	0.64	0.71
FL	0.71	0.64	0.75	0.74	0.73	0.57	0.73	0.78	0.71	0.70	0.77
DC	0.69	0.72	0.73	0.69	0.72	0.65	0.71	0.74	0.68	0.71	0.59
CA	0.68	0.74	0.57	0.63	0.61	0.69	0.78	0.52	0.67	0.79	0.77
**UT** a	0.68	0.68	0.74	0.66	0.59	0.68	0.77	0.75	0.63	0.55	0.73
NJ	0.67	0.74	0.67	0.82	0.56	0.69	0.48	0.71	0.60	0.62	0.85
RI	0.64	0.78	0.68	0.73	0.75	0.51	0.49	0.69	0.34	0.67	0.76
**OR**	0.62	0.79	0.58	0.59	0.73	0.63	0.44	0.68	0.79	0.56	0.43
**ID** a	0.62	0.65	0.74	0.62	0.63	0.75	0.61	0.30	0.63	0.69	0.53
**WY** a	0.59	0.69	0.57	0.55	0.59	0.71	0.67	0.43	0.65	0.43	0.64
DE	0.56	0.44	0.66	0.55	0.47	0.65	0.65	0.41	0.52	0.52	0.68
**MT** a	0.55	0.54	0.55	0.37	0.75	0.50	0.63	0.59	0.61	0.47	0.52
**ND** a	0.55	0.61	0.67	0.53	0.62	0.37	0.60	0.47	0.64	0.42	0.57
**NV** a	0.40	0.33	0.46	0.26	0.42	0.54	0.67	0.17	0.19	0.50	0.47

a Low workflow states.

The ten best common-fit parameter combinations (Table 2) were used for these hindcast projections. Results are ordered based on best average correlation (among the ten simulations for each state). The ten states with lowest 1972–2002 population density are shown in bold.

Overall, the cross-validation shows that the best combined-fit parameter combinations can simulate influenza seasonality throughout the country. Future use of higher resolution AH and observed P&I data that better represent local conditions may improve these model results.

### Additional SIRS Model Results

**Figure 4:**
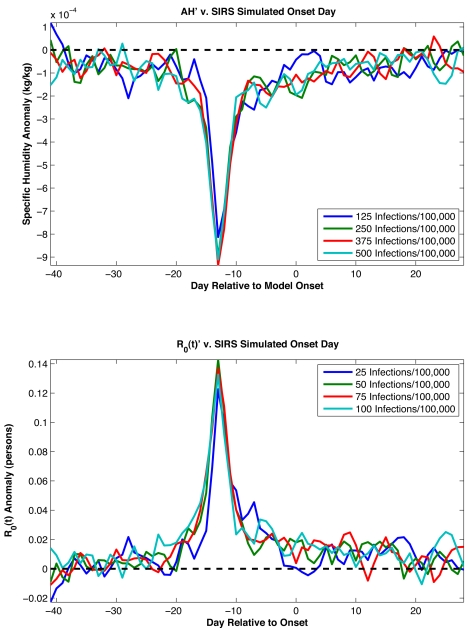
*AH′* associated with SIRS simulated influenza onset. Top, plots of average *AH′* associated with wintertime influenza onset for the ten best-fit SIRS model simulations at the five state sites (Arizona, Florida, Illinois, New York, and Washington). The onset dates are defined as the date on which wintertime infection rates have been at or above a prescribed level for two continuous weeks (e.g., 50 infections/100,000 people/day). Each solid line is the averaged *AH′* associated with influenza onset as defined by a different threshold infection rate. The dashed line shows *AH′* = 0. Bottom, plots of *R*_0_(*t*) anomalies using the *AH′* values. The *R*_0_(*t*) anomalies are calculated using the parameters *R*_0max_ and *R*_0min_ from each best-fit simulation (Table S3). The dashed line shows *R*_0_(*t*)**′** = 0.

We also used SIRS model simulations to provide additional support for the association between negative *AH′* and epidemic onset (Figure 2). Best-fit SIRS model runs reveal a comparable effect in which large negative *AH′* develop about 2 wk prior to onset as defined by SIRS model infection rates (Figure 4). The 1-wk difference in lag between this analysis with model infection rates (2 wk) and the analysis with observed excess P&I mortality rates (3 wk) roughly corresponds to the median time from infection to mortality [16]–[18]. The broader peak of negative *AH′* seen in Figure 2 is likely due to other, real-world factors that affect onset response and are not represented in the SIRS model (see Text S1).

Finally, we examined whether the school calendar, which alters person-to-person contact rates, could provide a better simulation of seasonal influenza than AH. School holidays have been estimated to lead to changes of ∼25% in influenza transmission [19] and occur during summer in the US, as well as at the end of the calendar year and again in spring. A number of SIRS model simulations were performed that included a stepwise increase of *R*_0_(*t*) during the school year (see Text S1). Simulations in which school closure was the only modulation of *R*_0_(*t*) were able to generate a winter seasonal cycle of influenza; however, these simulations did not reproduce observed excess P&I mortality as well as those with AH alone (see Figures S14 and S15; Table S5; Text S1). In addition, a 40%–90% change in influenza transmission (*R*_0_(*t*)) was needed to effect this seasonality (Table S5). This range of *R*_0_(*t*) changes is slightly larger than the previously estimated modulation of ∼25%; however, these previous estimates were derived from an age-structured population model, so direct comparison is difficult.

## Discussion

Distinguishing among potential environmental drivers of influenza seasonality, such as AH, RH, temperature, solar insolation, and the school calendar, is difficult since all demonstrate a similarly strong annual periodicity. Nevertheless, our findings indicate that AH is a major (and likely the predominant) determinant of influenza seasonality due to: 1) the empirical association of negative *AH′* with the onset of wintertime influenza outbreaks (Figure 2), which is statistically stronger than for RH, temperature or solar insolation (Table 1); 2) the relative consistency of the response to AH among the five states modeled in detail (i.e., similar parameter space; Table S2); and 3) the SIRS cross-validation showing that the same best-fit parameters (Table 2) can produce successful simulations of influenza seasonality throughout much of the US (Table 3).

In addition, several findings undermine the hypothesis that the association between the seasonal influenza cycle and AH is in fact due to confounding by other potential drivers. The case for solar insolation is weakened by its implausible positive association with wintertime influenza onset. Although laboratory analyses find that low RH favors influenza virus survival and transmission, RH is in fact typically incorrectly phased in the outdoor environment (i.e., maximal during winter, minimal during summer) and cannot explain peak wintertime influenza incidence. The case for temperature is weakened by the small amplitude of its seasonal cycle in most indoor environments. Finally, reanalyses of laboratory experiments indicate that AH is the best single-variable constraint of influenza virus survival and transmission [11]; associations with temperature and RH likely merely reflect their positive covariability with AH at various time scales. Still, a role for temperature or other (possibly multiple) covariable factors cannot be entirely discounted. Further laboratory investigation is needed to determine the effects of humidity, evaporation, and temperature on virus protein structure and survival.

SIRS model simulations also indicate that although the school calendar can explain seasonal epidemic influenza, the correspondence with observations is not as good as for simulations driven by AH (Figures S14 and S15). The required increase in transmissibility during school terms is greater than estimated previously; with such large variation in transmission, inclusion of non-summer breaks creates a noticeable decline in transmission in the Christmas and spring periods that is not observed in data (see Text S1). Nonetheless, an effect of school closure on influenza transmission rates is well documented [19],[20] and cannot be discounted. It is certainly possible that the effects of AH and the school calendar on influenza transmission act in concert with one another; however, our statistical and SIRS model findings indicate that AH variability provides a more parsimonious explanation for the seasonality of epidemic influenza in temperate regions, and in addition, is associated with the onset date of individual wintertime outbreaks. The argument that AH at least partly determines influenza seasonality is supported by: 1) laboratory evidence [11]; 2) the much weaker seasonality in the tropics where humidity is high year-round, but a school calendar exists; 3) the *AH′*-onset analysis (Figures 2 and 4); 4) the plausibility of parameter combinations and the effect size for AH within SIRS model simulations (Figures 1 and 3; Table 2); and 5) the superior overall quality of AH-forced simulations (Figure S14) and their reduced sensitivity to stochastic processes within the SIRS model (Figure S15).

There are minor differences among the sites in the best-fit parameter values for the SIRS model (Figures S7, S8, S9, S10, and S11, and Table S2), some of which could be host mediated. For instance, Florida and New York show a tendency toward lower duration of immunity. This difference could be derived from a number of host-mediated factors specific to these states. The findings presented here do not preclude an influence of such factors on influenza transmission and seasonality. Differences in population susceptibility and infectivity (e.g., population age and general health), seasonal variations of host behavior (e.g., more time indoors in close contact during winter [19]), and host resistance (e.g., wintertime melatonin or vitamin D deficiencies [12],[13]) may still affect influenza transmission rates.

Among states, there are also differences of average peak SIRS-simulated *R*_E_(*t*) (Figure 1F); however, there is no systematic relationship between rates of observed excess P&I deaths and those peak *R*_E_(*t*) values among these sites. For instance, Florida and New York have similar rates of observed excess mortality per 100,000 persons, but different peak *R*_E_(*t*) levels. State-to-state differences in contact rates and population age and structure, in particular the proportion of seniors, who are at highest risk of influenza-related death during seasonal epidemics, undoubtedly affect influenza infection and mortality rates, and modulate the amplitude and duration of individual outbreaks. In addition, the dominant influenza subtype is a key predictor of influenza-related mortality rate each season; A/H3N2-dominant seasons are associated with two to three times higher death rates than H1N1 and B-dominant seasons [3],[4]. These other factors are not accounted for in our SIRS model; hence, there is not a good one-to-one correspondence between the average peak size of *R*_E_(*t*) and rates of observed excess P&I deaths. However, within the SIRS model, a relationship between *R*_E_(*t*) and simulated infection rates does exist. Among the ten best-fit simulations at each site, the average maximum *R*_E_(*t*) rank (from greatest to least) as New York, Illinois, Washington, Arizona, Florida. Similarly among these runs, the average maximum epidemic size ranks (from greatest to least) as New York, Washington, Illinois, Arizona, Florida. This more direct response is not unexpected; within the SIRS model, higher *R*_E_(*t*) directly corresponds to greater transmission and, consequently, more rapidly developing, larger outbreaks.

It should be noted that observed excess P&I mortality is an imperfect indicator of influenza incidence, as other respiratory illnesses exhibit similar seasonal periodicities. No doubt these other diseases contribute to the seasonality of the observational time series used here (Figure 3, Figure S1). However, excess P&I mortality generally shows a strong correspondence with other indicators of influenza incidence, such as hospitalization data and laboratory notifications [4]. A clearer picture of the environmental determinants of influenza seasonality and onset will emerge when the effects of AH and other environmental variables on these potentially confounding, seasonal respiratory pathogens are also elucidated.

The initial evidence demonstrating that AH affects influenza virus survival and transmission was derived from laboratory experiments studying the airborne transmission of influenza; however, our SIRS model uses no specific mode of transmission. Thus, other modes of transmission, in particular indirect transmission via fomites, if similarly affected by AH, might also have a role determining the seasonality of influenza in temperate regions. In addition, the SIRS model is highly idealized and fails to represent many factors in the real world that can affect transmission rates, including clustered populations, structured interactions, variation in host infectiousness, and multiple influenza strains conferring various levels of cross-immunity. Future work could incorporate these effects into a more realistic influenza model that also accounts for the effects of AH. Such efforts would also enable better discrimination between the effects of school terms and AH. Also, the effects of AH on influenza transmission should be incorporated into models accounting for travel and workflow [4],[21],[22] to explain the seasonal geographic spread of influenza.

The analyses presented here need to be extended elsewhere, including the tropics, where AH is high year-round and the seasonality of influenza is often less clearly defined. High AH does not preclude but merely reduces influenza virus survival and transmission, so it is possible a role for AH also exists in the tropics. However, the findings presented here suggest that *R*_0_(*t*) would be less sensitive to AH variability in areas of very high year-round AH, such as the tropics, which may allow for other, possibly host-mediated, factors to play a more predominant role in generating seasonal variability in influenza incidence.

Laboratory studies provided the initial evidence that AH may determine the seasonality of influenza in temperate regions [11]. The model and statistical results presented here indicate that the effects of AH observed in the laboratory are sufficient to explain patterns observed at the population level and illustrate the power of epidemiological modeling rooted in individual-level experiments. The results indicate that AH affects both the seasonality of influenza incidence and the timing of individual wintertime influenza outbreaks in temperate regions. The association of negative *AH′* with wintertime influenza outbreak onset is remarkable given the noise in the data and suggests that skillful, short-term probabilistic forecasts of epidemic influenza could be developed.

## Methods

### SIRS Model and Methods

The SIRS model equations are:
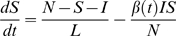
(2)

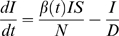
(3)where *S* is the number of susceptible people in the population, *t* is time in years, *N* is the population size, *I* is the number of infectious people, *N*−*S*−*I* is the number of resistant individuals, *β*(*t*) is the contact rate at time *t*, *L* is the average duration of immunity in years, and *D* is the mean infectious period in years. The basic reproductive number at time *t* is *R*_0_(*t*) = *β*(*t*)*D*.

Observed AH conditions were derived from National Centers for Environmental Prediction–National Center for Atmospheric Research (NCEP-NCAR) reanalysis [23]. For each state (Arizona, Florida, Illinois, New York, and Washington), a daily 1972–2002 time series of 2-m above-ground specific humidity, *q*(*t*), was constructed by averaging all grid cells with ≥10% of their area within that state. The equation relating *q*(*t*) to *R*_0_(*t*) uses an exponential functional form similar to the relationships between AH and both influenza virus survival and transmission, derived from laboratory experiments (Figure 1):
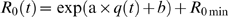
(4)where *a* = −180, *b* = log(*R*_0max_−*R*_0min_), *R*_0max_ is the maximum daily basic reproductive number, and *R*_0min_ is the minimum daily basic reproductive number. The value of *a* is estimated from the laboratory regression of influenza virus survival upon AH (Figure 1A). Equation 4 dictates that *R*_0_(*t*) = *R*_0max_ when *q*(*t*) = 0 kg/kg and that *R*_0_(*t*) approaches *R*_0min_ asymptotically as *q*(*t*) increases.

For simulations, we use a stochastic Markov chain formulation in which individuals are treated as discrete entities, and transitions between model states (i.e., susceptible, infected, recovered) are determined by random draws corresponding to rates determined from Equations 2 and 3. Using the daily time series of *q*(*t*), which alters *R*_0_(*t*), daily influenza transmission was simulated for each of the five state sites during 1972–2002 for a model population of 500,000 individuals. Initial conditions included 50,000 susceptible persons and 100 infected persons; however, results were not sensitive to these numbers. Simulations were performed with daily random seeding of infected individuals (i.e., each day there is a 10% probability that a single susceptible individual becomes infected), meant to represent reintroduction of the virus in the model domain due to travel. The model is perfectly mixed and simulations were performed with two influenza virus subtypes: A-H1N1/B and A-H3N2 (see Figures S12 and S13). No cross immunity was conferred within the model between these virus subtypes. Each year, beginning in May, the random seeding of infectious individuals in the population (representing emigration/travel) is fixed to the dominant recorded subtype for the US (i.e., either A-H1N1/B or A-H3N2), based on Centers for Disease Control/Morbidity and Mortality Weekly Report (CDC/MMWR) laboratory and antigenic surveillance data [3],[4].

Five thousand simulations at each site were run using combinations of the four model parameters: *R*_0max_, *R*_0min_, *D*, and *L* chosen using a Latin hypercube sampling structure with uniform distribution. The ranges of these parameters were specified to reflect known influenza dynamics (see Table S4, Text S1). Estimates of *R*_0_ derived or used by many authors range from 1.3 to 3 [16],[21],[22],[24]–[27]. To effect this range, given Equation 3 and variations of *q*(*t*), we used an *R*_0max_ ranging from 1.3 to 4. *R*_0min_ provides the *R*_0_ below which the model cannot fall. This minimum recognizes that other modes of influenza transmission exist that may not be modulated by absolute humidity. *R*_0min_ values range from 0.8 to 1.3. Per Equation 4, decreasing humidity increases *R*_0_(*t*). The range of this nonlinear increase is set by the randomly chosen *R*_0max_ and *R*_0min_ parameters. Estimates of *D* range from 2 to 7 d [16],[25], and estimates of *L* range from 2 to 10 y. Both influenza virus subtypes used the same four randomly chosen parameters during each simulation, though results were similar when each subtype was assigned different parameters (eight in total).

The quality of each simulation at each site was evaluated based on RMS error with observed excess P&I mortality [3],[4] (Figure S1), lagged 2 wk. The lag accounts for mean time from infection to mortality. Prior to determining the RMS error, each model run was scaled to enable comparison of simulated infections with observed mortality rates (see Text S1).

Prior to the model cross-validation throughout the contiguous US, we first determined the effect that stochasticity within each SIRS simulation has on the quality of fit with observations. We reran the ten best common-fit parameter combinations 100 times each for New York, each time with a different random seeding, and found that correlations to observed P&I mortality ranged from *r* = 0.65 to *r* = 0.97 with an average of *r* = 0.87. In contrast, multiple simulations with the 4,500th best parameter combination, out of 5,000, produced correlations that ranged from *r* = −0.18 to *r* = 0.34. Thus, random seeding within a particular model run produces a range of correlation coefficient outcomes; however, good model runs should produce high positive correlation with observations (average *r*>0.7, minimum *r*>0.5).

## Supporting Information

Figure S1 **Observed P&I mortality per 100,000 people in the US, 1972–2002.** (A) Time series of weekly P&I mortality (blue line). Note the winter seasonal mortality peaks each winter. The dotted vertical lines denote the first week of January of each year, and the red curve is a seasonal baseline representing the expected P&I mortality in the absence of influenza. (B) A robust indicator of the timing and impact of influenza epidemics is excess P&I mortality (black line), which measures mortality attributable to influenza above the seasonal baseline.
(0.15 MB PDF)

Figure S2 **Plots of** ***AH′*** **averaged for the 6 wk prior and 4 wk following the onset of wintertime influenza for three regions within the US.** The three regions are as follows: top, the Southwest (Arizona, Colorado, Nevada, New Mexico, and Utah); middle, the Northeast (Connecticut, the District of Columbia, Delaware, Maine, Maryland, Massachusetts, New Hampshire, New Jersey, New York, Pennsylvania, Rhode Island, Vermont, and West Virginia); and bottom, the Gulf region (Alabama, Arkansas, Florida, Georgia, Kentucky, Louisiana, Mississippi, North Carolina, South Carolina, Tennessee, and Virginia). The onset dates are defined as the date at which wintertime observed excess P&I mortality had been at or above a prescribed threshold level for two continuous weeks (e.g., 0.01 deaths/100,000 people). Each solid line is the averaged *AH′* associated with influenza onset as defined by a different threshold mortality rate. The dashed line shows *AH′* = 0. Both Texas and California were excluded from these regional analyses due to their large geographic size, which span a large range of AH conditions.
(0.31 MB GIF)

Figure S3 **Plots of** ***AH′*** **averaged for the 6 wk prior and 4 wk following the onset of wintertime influenza for three additional regions within the US.** As for Figure S2, but for: top, the Northwest (Idaho, Montana, Oregon, and Washington); middle, the Great Lakes region (Illinois, Indiana, Iowa, Michigan, Minnesota, Missouri, Ohio, and Wisconsin); and bottom, the Plains (Kansas, Nebraska, North Dakota, South Dakota, and Wyoming).
(0.30 MB GIF)

Figure S4 **Map of regional state groupings used in the analyses presented in Figures S2 and S3.** Southwest states are in red; Northeast states are in blue; Gulf states are in green; Pacific Northwest states are in cyan; Great Lakes states are in yellow; and Plains states are in magenta. California, Oklahoma, and Texas were not included in any region, but were used in the analysis performed for the contiguous US (Figure 2; Table 1).
(0.03 MB GIF)

Figure S5 **Plots of SIRS model–simulated infections for individual years from the best-fit simulation in New York state.**
(0.09 MB EPS)

Figure S6 **Plots of RMS error of the 5,000 SIRS dual-strain simulations as a function of parameter space.** Shown are the RMS error based on combined simulation fit at all five sites in aggregate (the same parameter combinations were run at all five sites).
(1.50 MB GIF)

Figure S7 **Plots of RMS error of the 5,000 SIRS dual-strain simulations at Arizona as a function of parameter space.**
(1.50 MB GIF)

Figure S8 **Plots of RMS error of the 5,000 SIRS dual-strain simulations at Florida as a function of parameter space.**
(1.49 MB GIF)

Figure S9 **Plots of RMS error of the 5,000 SIRS dual-strain simulations at Illinois as a function of parameter space.**
(1.52 MB GIF)

Figure S10 **Plots of RMS error of the 5,000 SIRS dual-strain simulations at New York state as a function of parameter space.**
(1.52 MB GIF)

Figure S11 **Plots of RMS error of the 5,000 SIRS dual-strain simulations at Washington state as a function of parameter space.**
(1.48 MB GIF)

Figure S12 **Power spectra of the third best-fit New York single-strain SIRS simulation (top) and the New York observed excess P&I mortality data (bottom), shown for 1975–2002.** Harmonic 28 gives the power at 1-y period; harmonic 56 gives the power at 6-mo period.
(0.06 MB GIF)

Figure S13 **Power spectra of the tenth best-fit New York dual-strain SIRS simulation.** Power spectra of a typical (tenth) best-fit New York dual-strain discrete SIRS simulation (top), broken down by subtype (middle two panels), and the New York observed excess P&I mortality data (bottom), 1975–2002.
(0.12 MB GIF)

Figure S14 **Histograms of correlation coefficients for SIRS model ensemble simulations in New York state.** Each distribution presents the correlation coefficients for 5,000 simulations, each run with a different parameter combination, for a given model forcing. Shown are results for simulation with school forcing (no weekends or holidays), school forcing (with weekends and holidays), AH forcing, and combined AH and school forcing. For the combined AH and school forcing, the school forcing did not represent weekends and holidays. Correlations are with 1972–2002 New York state observed excess P&I mortality.
(0.03 MB EPS)

Figure S15 **Test of the effect of stochasticity within the SIRS model on well-matched simulations in the state of New York.** The ten best-fit parameter combinations for the AH-only (Table S2) and school-only (no breaks; Table S5) simulations were each run an additional 100 times, each time with a different random seeding. Histograms of correlations with 1972–2002 New York state observed excess P&I mortality are shown.
(0.01 MB EPS)

Table S1 **Correlation coefficients of daily anomalies in wintertime (October–February) surface meteorological variables for the lower 48 US states and DC across all these sites, 1972–2002.**
(0.03 MB DOC)

Table S2 **Parameter combinations for the ten best-fit dual-strain SIRS simulations at each site with the parameters** *R*_0max_ **,** *R*_0min_ **,** ***D*** **, and** ***L*** **randomly chosen from within specified ranges.** Best-fit simulations were selected based on RMS error after scaling the 31-y mean daily infection number to the 31-y mean observed daily excess P&I mortality rate. The scaling factor and correlation with observed mean annual excess P&I mortality rates are also shown.
(0.13 MB DOC)

Table S3 **Parameter combinations for the ten best-fit simulations at the Arizona, Florida, Illinois New York, and Washington state sites.** Five thousand simulations were performed at each site with *D* = 2.4 d and the three remaining parameters randomly chosen from the ranges: *L* = 2–8 y, *R*_0max_ = 2–4, and *R*_0min_ = 1–1.3. Best-fit simulations were selected for the five sites in aggregate based on RMS error after scaling the 31-y mean daily infection number to the 31-y mean observed daily excess P&I mortality rate at each site. The scaling factor itself, representing mortality per infection, is also shown.
(0.05 MB DOC)

Table S4 **Comparison of best common-fit model simulation (** **Table 1** **) parameter fluctuations at the five sites with those of Dushoff et al., 2004.**
(0.07 MB DOC)

Table S5 **Parameter combinations for the ten best-fit simulations using only the school calendar at the New York state site.** Five thousand simulations were performed with the parameters *SC*, *R*_0min_, *D*, and *L* randomly chosen from within specified ranges. Best-fit simulations were selected based on RMS error after scaling the 31-y mean daily infection number to the 31-y mean observed daily excess P&I mortality rate.
(0.04 MB DOC)

Text S1 **Supporting text providing additional descriptions of methodologies and findings.**
(0.17 MB DOC)

## Abbreviations

AH: absolute humidity
P&I: pneumonia and influenza
RH: relative humidity
RMS: root mean square
