# Correction: Absolute Humidity and the Seasonal Onset of Influenza in the Continental United States

**DOI:** 10.1371/annotation/9ddc5251-72a1-4eba-ae35-9ab04488527b

**Published:** 2010-03-15

**Authors:** Jeffrey Shaman, Virginia E. Pitzer, Cécile Viboud, Bryan T. Grenfell, Marc Lipsitch

The authors declare the following competing interest, which should have been declared at the time of publication. M.L. discloses consulting income from the Avian/Pandemic Flu Registry (Outcome Sciences, funded in part by Roche) and from Novartis Vaccines and Diagnostics. The other authors declare no competing interests.

