# Correction: Absolute Humidity and the Seasonal Onset of Influenza in the Continental United States

**DOI:** 10.1371/annotation/35686514-b7a9-4f65-9663-7baefc0d63c0

**Published:** 2010-03-18

**Authors:** Jeffrey Shaman, Virginia E. Pitzer, Cécile Viboud, Bryan T. Grenfell, Marc Lipsitch

In the Funding section, the second grant number from the NIH Models of Infectious Disease Agent Study program was incorrect. The correct grant number is: 1U54GM088558. 

