# Supplementary figures and images for: Absolute Humidity and the Seasonal Onset of Influenza in the Continental United States
Source: PLoS Biol. 2010 Feb 23;8(2):e1000316. doi: 10.1371/journal.pbio.1000316 (PMC2826374; doi:10.1371/journal.pbio.1000316)

**A) Weekly P&I mortality rate time series, USA**

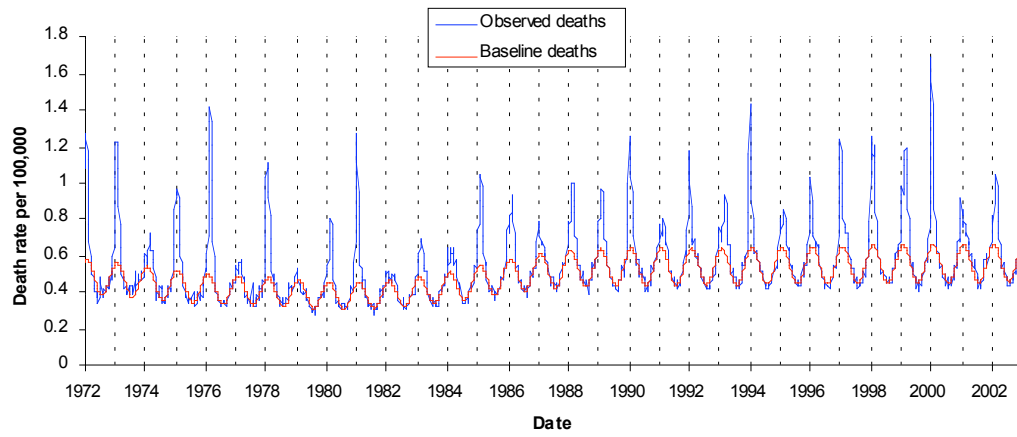

**B) Weekly excess P&I mortality rate time series, USA**

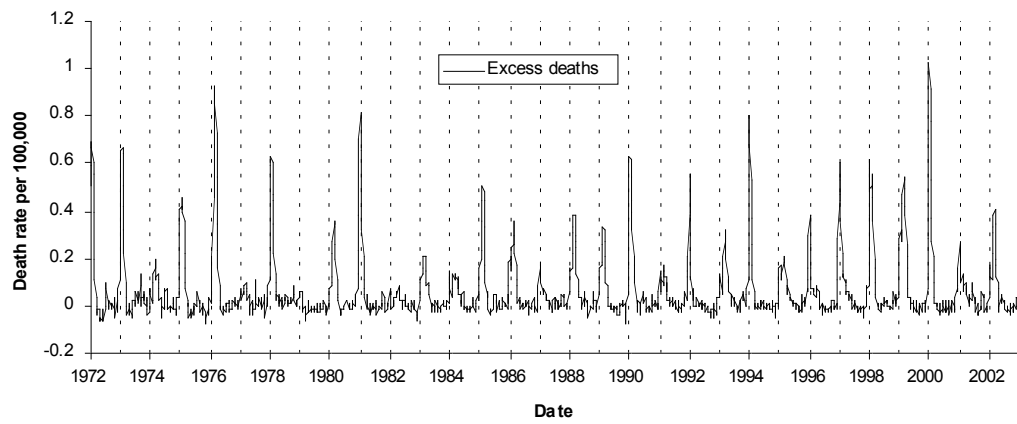

Supplement: Figure S1 — Observed P&I mortality per 100,000 people in the US, 1972–2002. (A) Time series of weekly P&I mortality (blue line). Note the winter seasonal mortality peaks each winter. The dotted vertical lines denote the first week of January of each year, and the red curve is a seasonal baseline representing the expected P&I mortality in the absence of influenza. (B) A robust indicator of the timing and impact of influenza epidemics is excess P&I mortality (black line), which measures mortality attributable to influenza above the seasonal baseline. (0.15 MB PDF) [file pbio.1000316.s001.pdf]

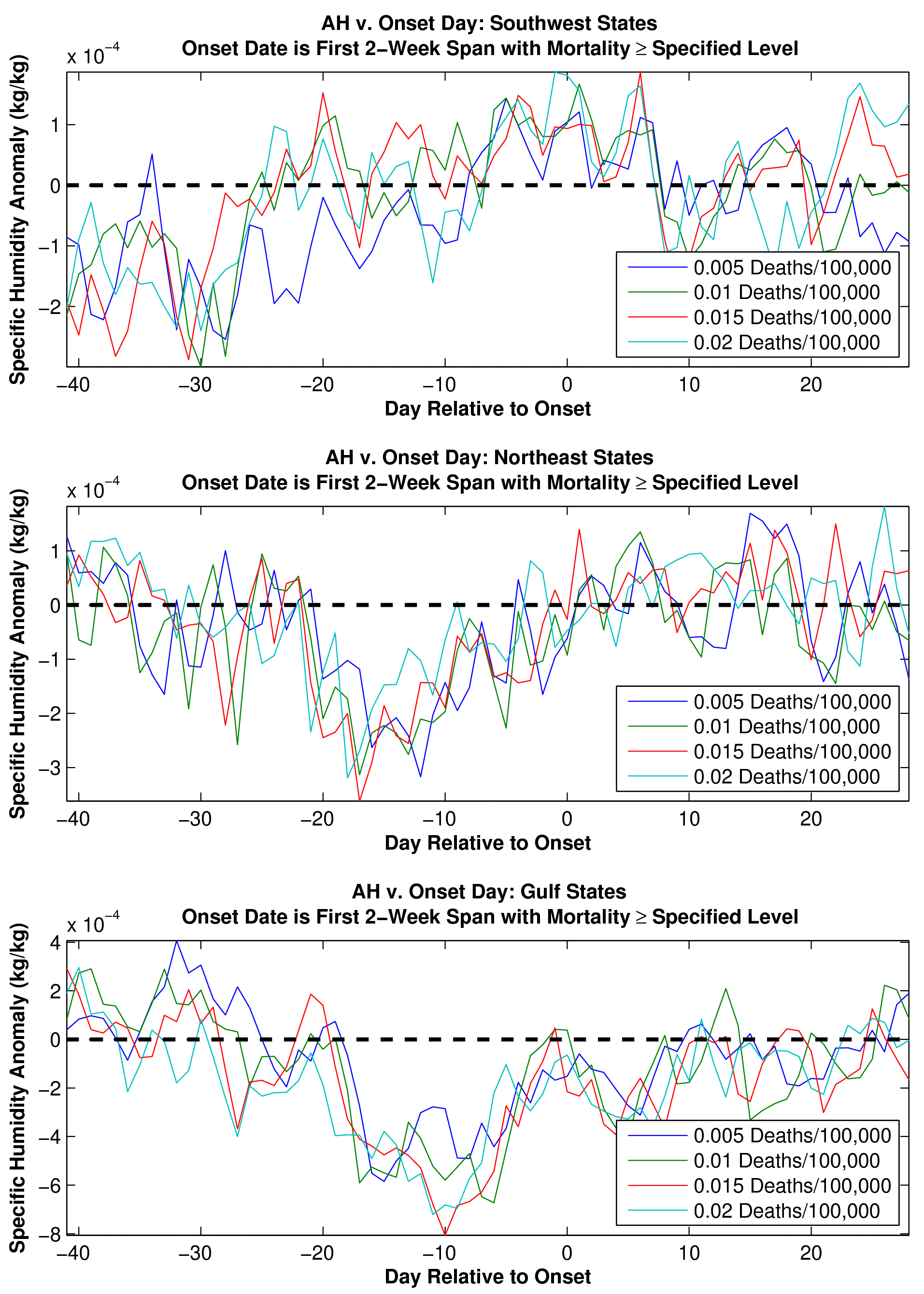

Supplement: Figure S2 — Plots of AH′ averaged for the 6 wk prior and 4 wk following the onset of wintertime influenza for three regions within the US. The three regions are as follows: top, the Southwest (Arizona, Colorado, Nevada, New Mexico, and Utah); middle, the Northeast (Connecticut, the District of Columbia, Delaware, Maine, Maryland, Massachusetts, New Hampshire, New Jersey, New York, Pennsylvania, Rhode Island, Vermont, and West Virginia); and bottom, the Gulf region (Alabama, Arkansas, Florida, Georgia, Kentucky, Louisiana, Mississippi, North Carolina, South Carolina, Tennessee, and Virginia). The onset dates are defined as the date at which wintertime observed excess P&I mortality had been at or above a prescribed threshold level for two continuous weeks (e.g., 0.01 deaths/100,000 people). Each solid line is the averaged AH′ associated with influenza onset as defined by a different threshold mortality rate. The dashed line shows AH′ = 0. Both Texas and California were excluded from these regional analyses due to their large geographic size, which span a large range of AH conditions. (0.31 MB GIF) [file pbio.1000316.s002.gif]

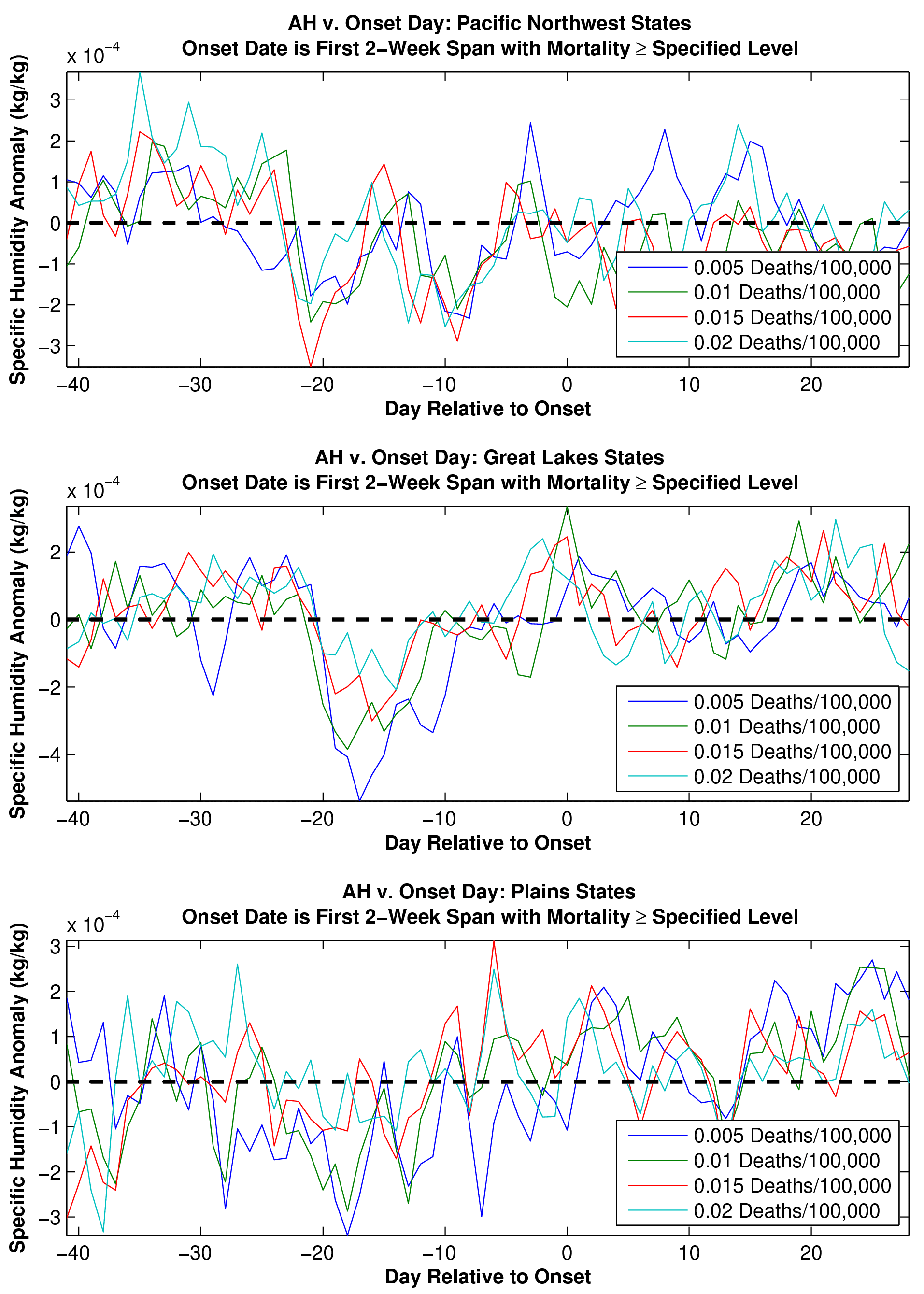

Supplement: Figure S3 — Plots of AH′ averaged for the 6 wk prior and 4 wk following the onset of wintertime influenza for three additional regions within the US. As for Figure S2, but for: top, the Northwest (Idaho, Montana, Oregon, and Washington); middle, the Great Lakes region (Illinois, Indiana, Iowa, Michigan, Minnesota, Missouri, Ohio, and Wisconsin); and bottom, the Plains (Kansas, Nebraska, North Dakota, South Dakota, and Wyoming). (0.30 MB GIF) [file pbio.1000316.s003.gif]

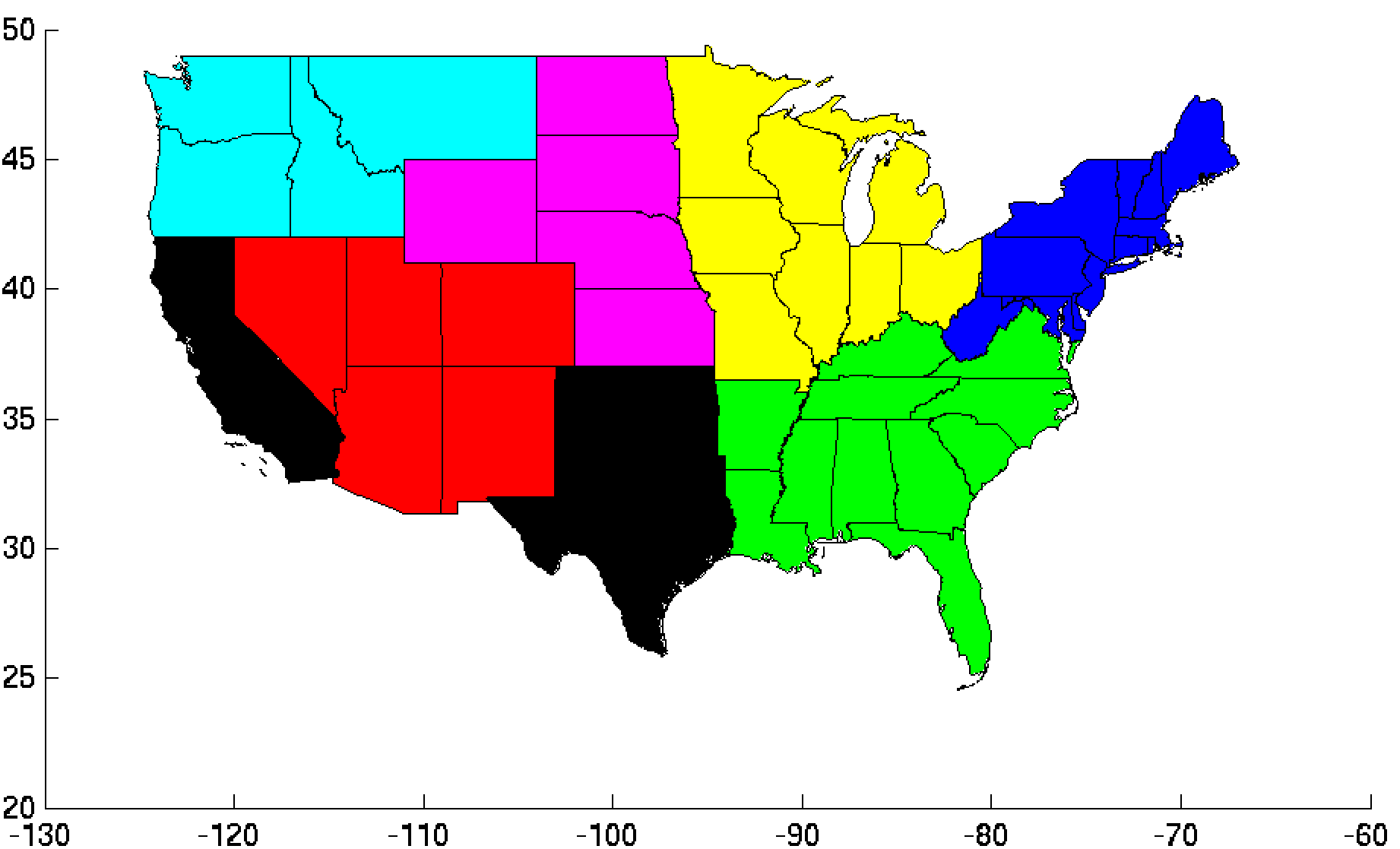

Supplement: Figure S4 — Map of regional state groupings used in the analyses presented in Figures S2 and S3. Southwest states are in red; Northeast states are in blue; Gulf states are in green; Pacific Northwest states are in cyan; Great Lakes states are in yellow; and Plains states are in magenta. California, Oklahoma, and Texas were not included in any region, but were used in the analysis performed for the contiguous US (Figure 2; Table 1). (0.03 MB GIF) [file pbio.1000316.s004.gif]

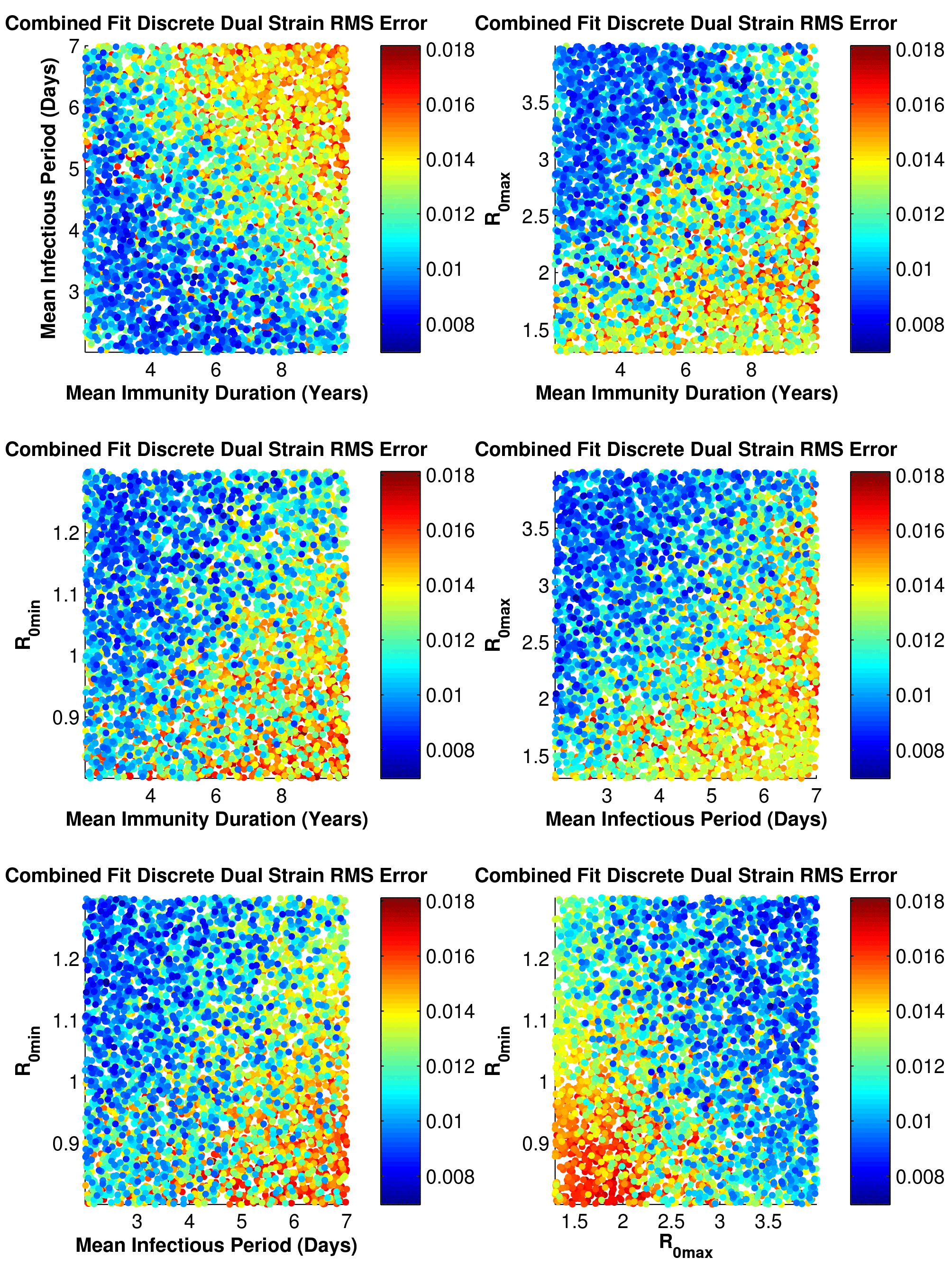

Supplement: Figure S6 — Plots of RMS error of the 5,000 SIRS dual-strain simulations as a function of parameter space. Shown are the RMS error based on combined simulation fit at all five sites in aggregate (the same parameter combinations were run at all five sites). (1.50 MB GIF) [file pbio.1000316.s006.gif]

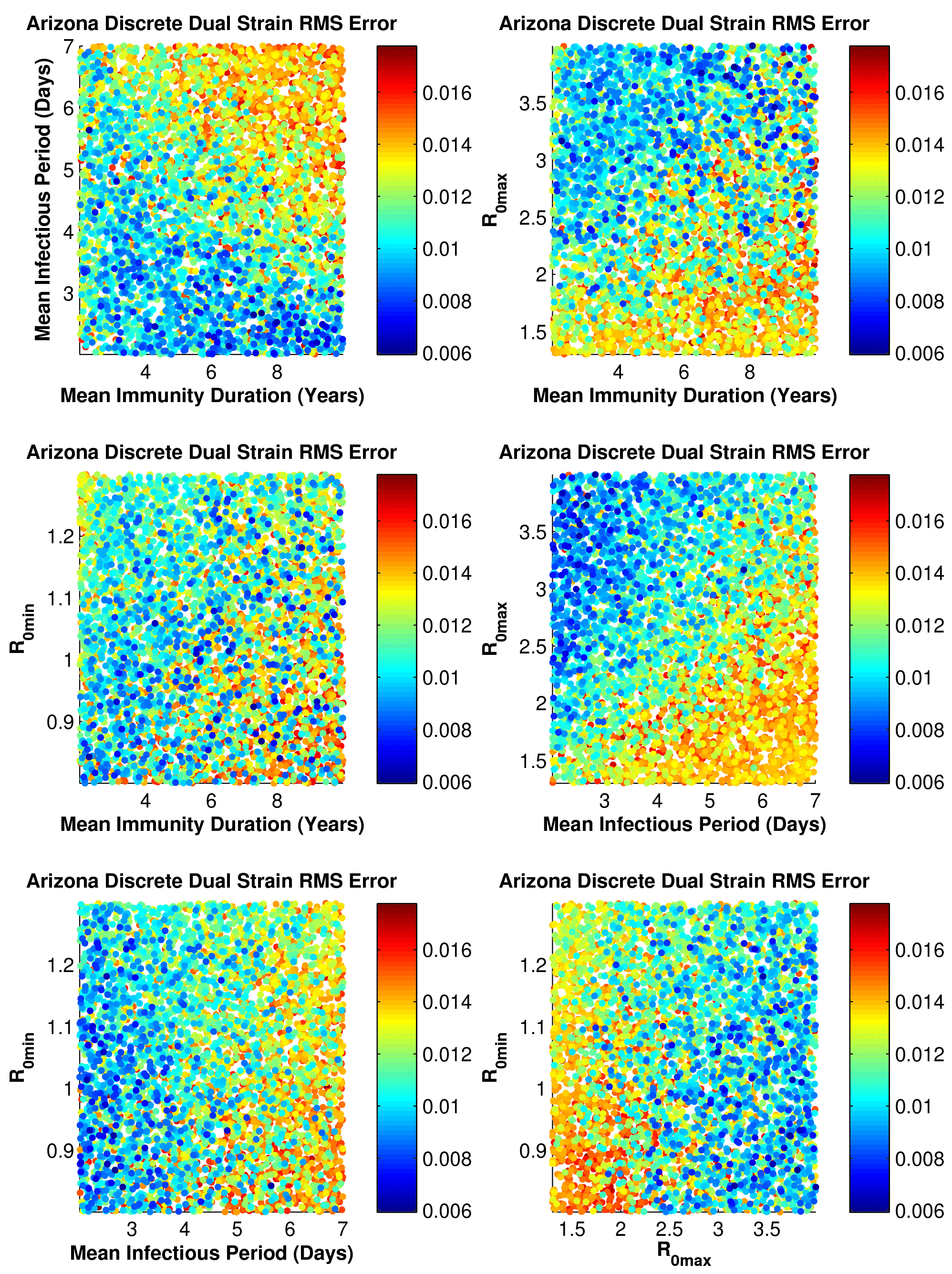

Supplement: Figure S7 — Plots of RMS error of the 5,000 SIRS dual-strain simulations at Arizona as a function of parameter space. (1.50 MB GIF) [file pbio.1000316.s007.gif]

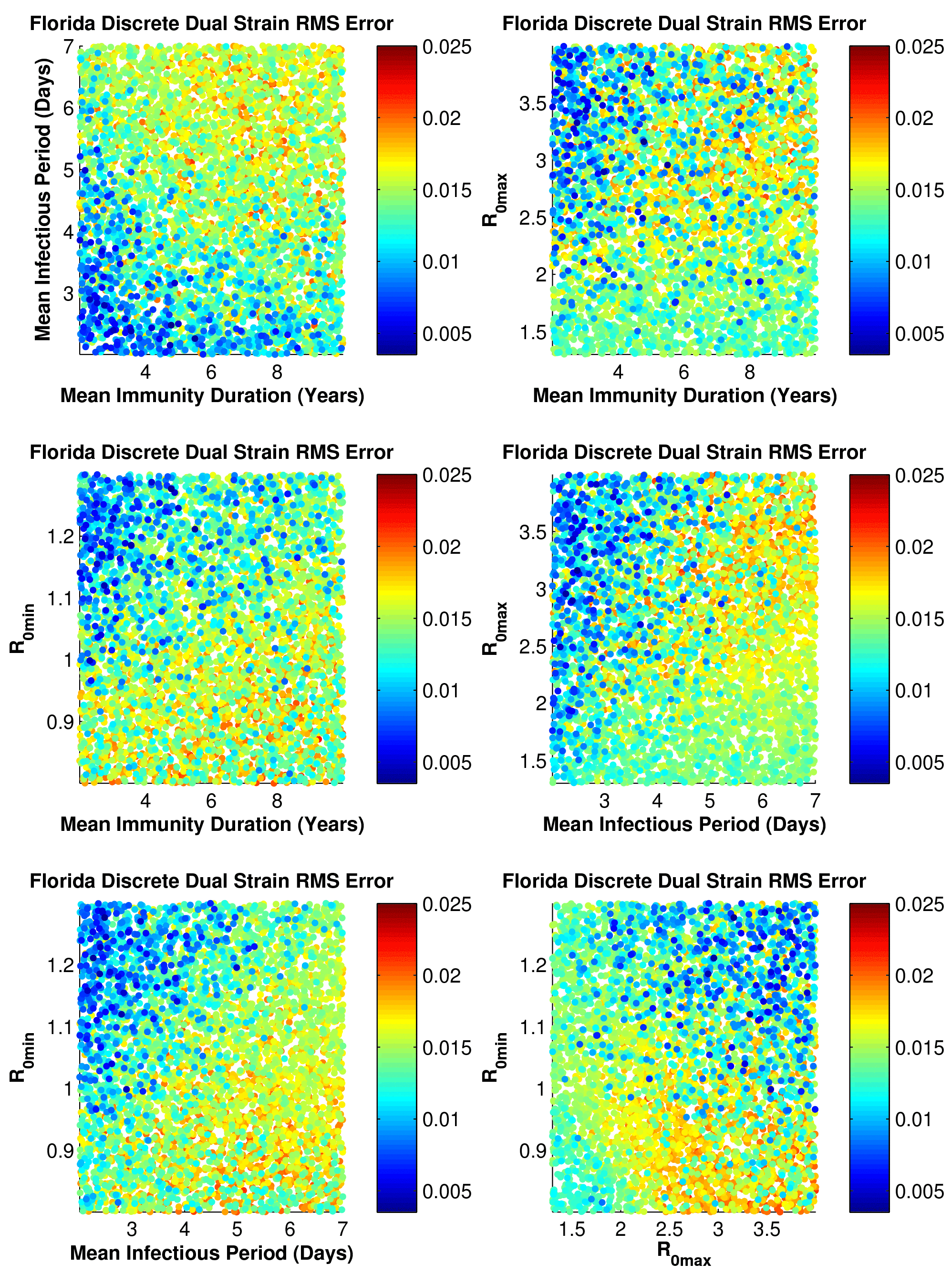

Supplement: Figure S8 — Plots of RMS error of the 5,000 SIRS dual-strain simulations at Florida as a function of parameter space. (1.49 MB GIF) [file pbio.1000316.s008.gif]

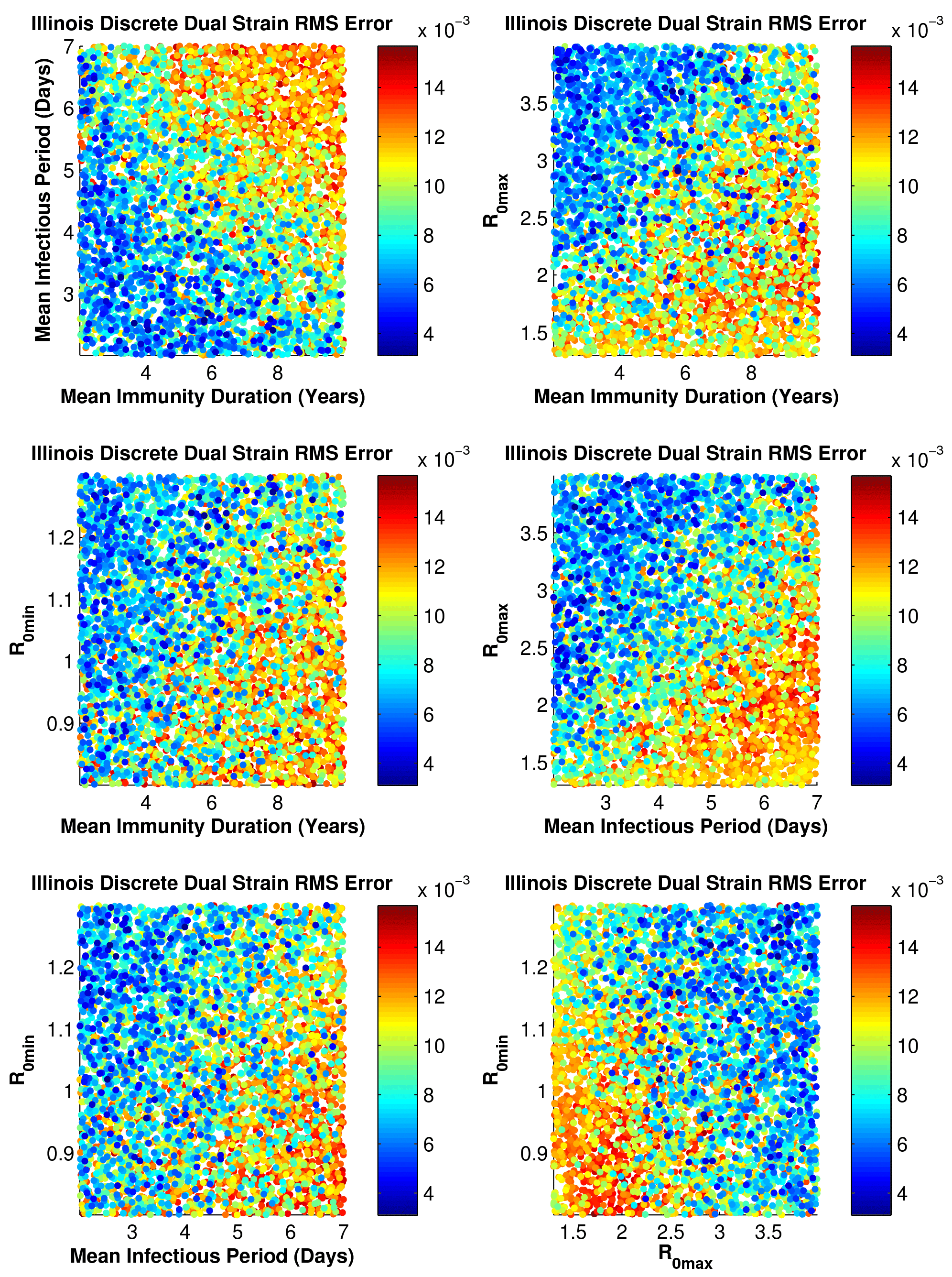

Supplement: Figure S9 — Plots of RMS error of the 5,000 SIRS dual-strain simulations at Illinois as a function of parameter space. (1.52 MB GIF) [file pbio.1000316.s009.gif]

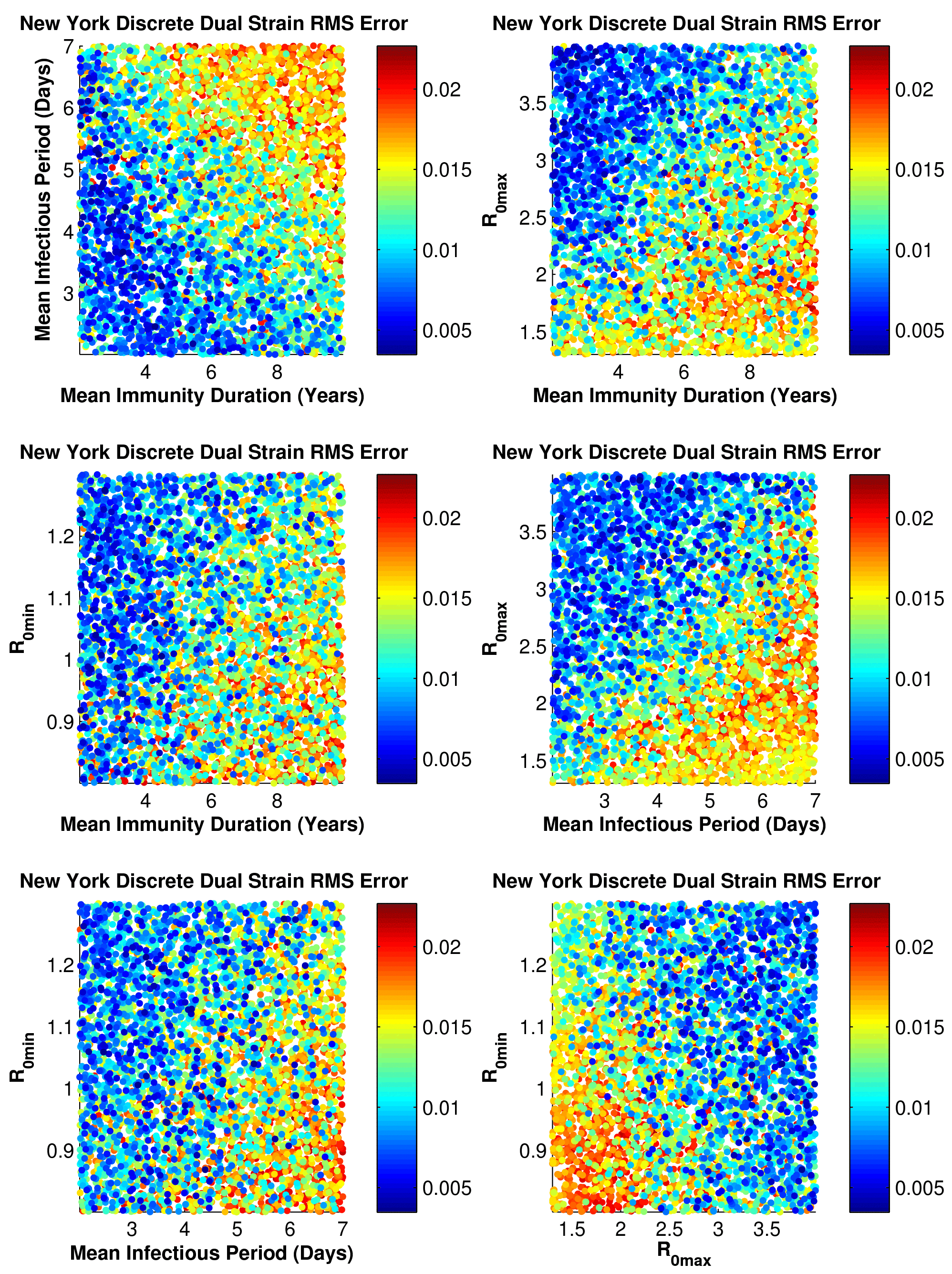

Supplement: Figure S10 — Plots of RMS error of the 5,000 SIRS dual-strain simulations at New York state as a function of parameter space. (1.52 MB GIF) [file pbio.1000316.s010.gif]

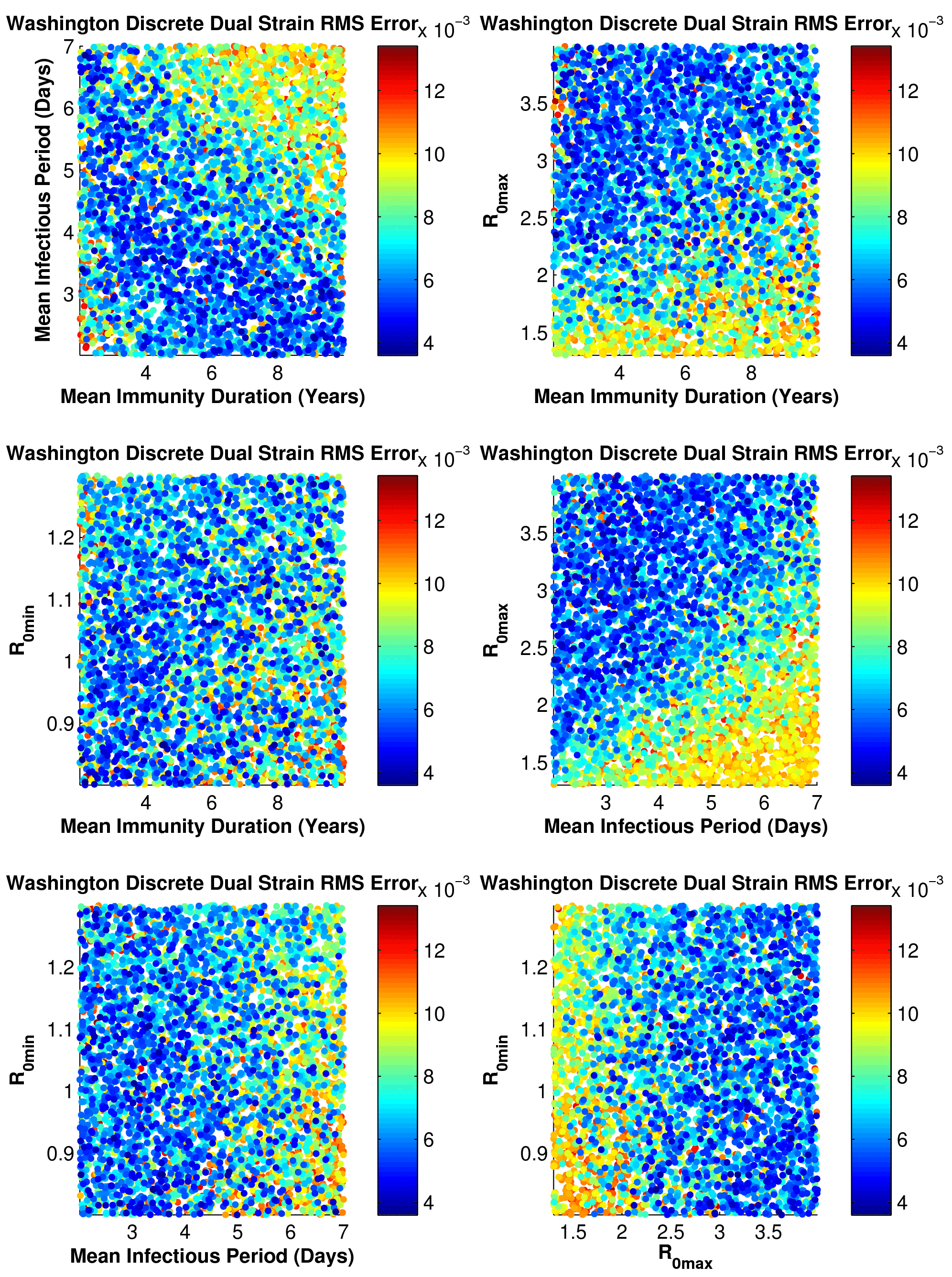

Supplement: Figure S11 — Plots of RMS error of the 5,000 SIRS dual-strain simulations at Washington state as a function of parameter space. (1.48 MB GIF) [file pbio.1000316.s011.gif]

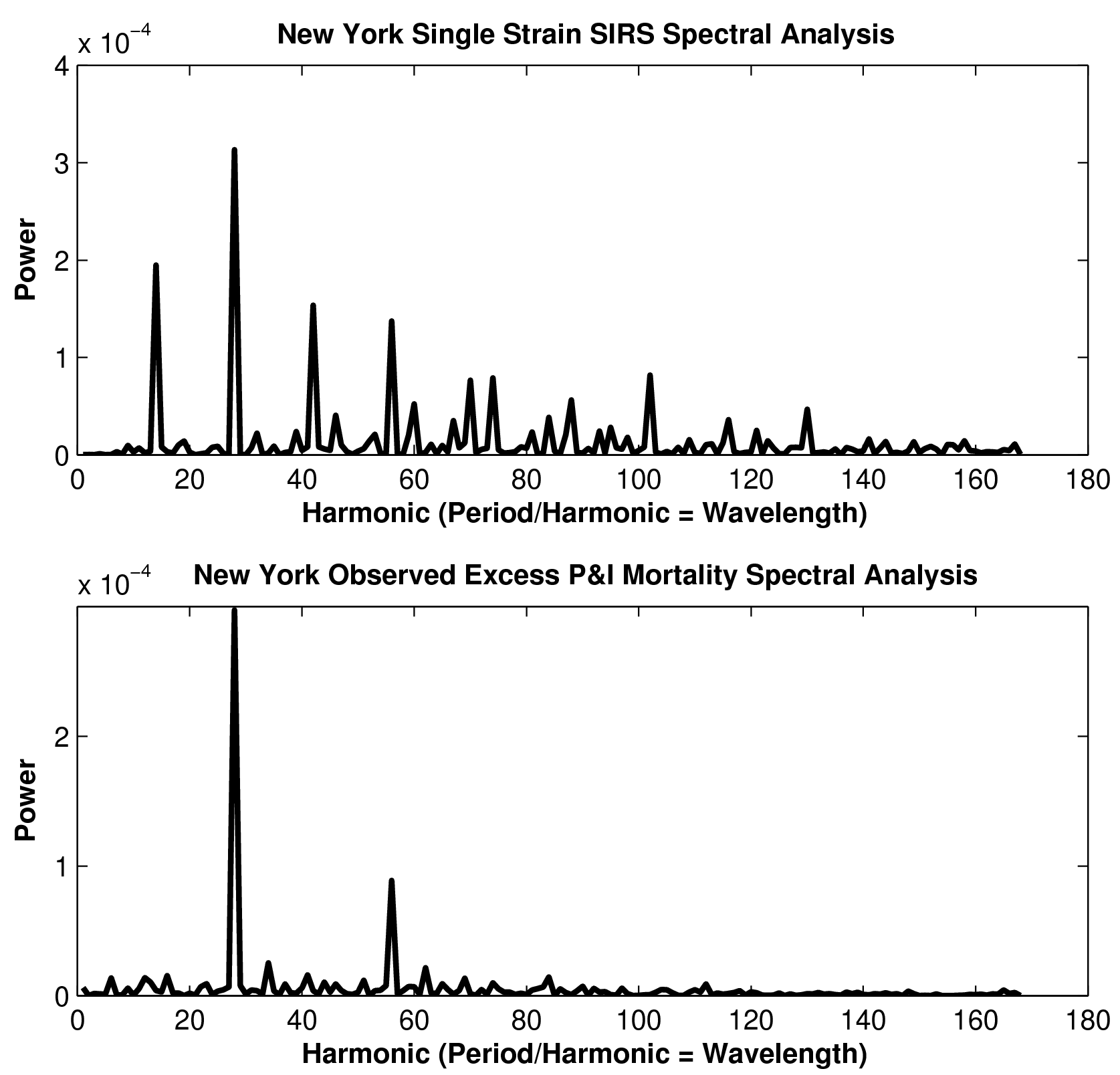

Supplement: Figure S12 — Power spectra of the third best-fit New York single-strain SIRS simulation (top) and the New York observed excess P&I mortality data (bottom), shown for 1975–2002. Harmonic 28 gives the power at 1-y period; harmonic 56 gives the power at 6-mo period. (0.06 MB GIF) [file pbio.1000316.s012.gif]

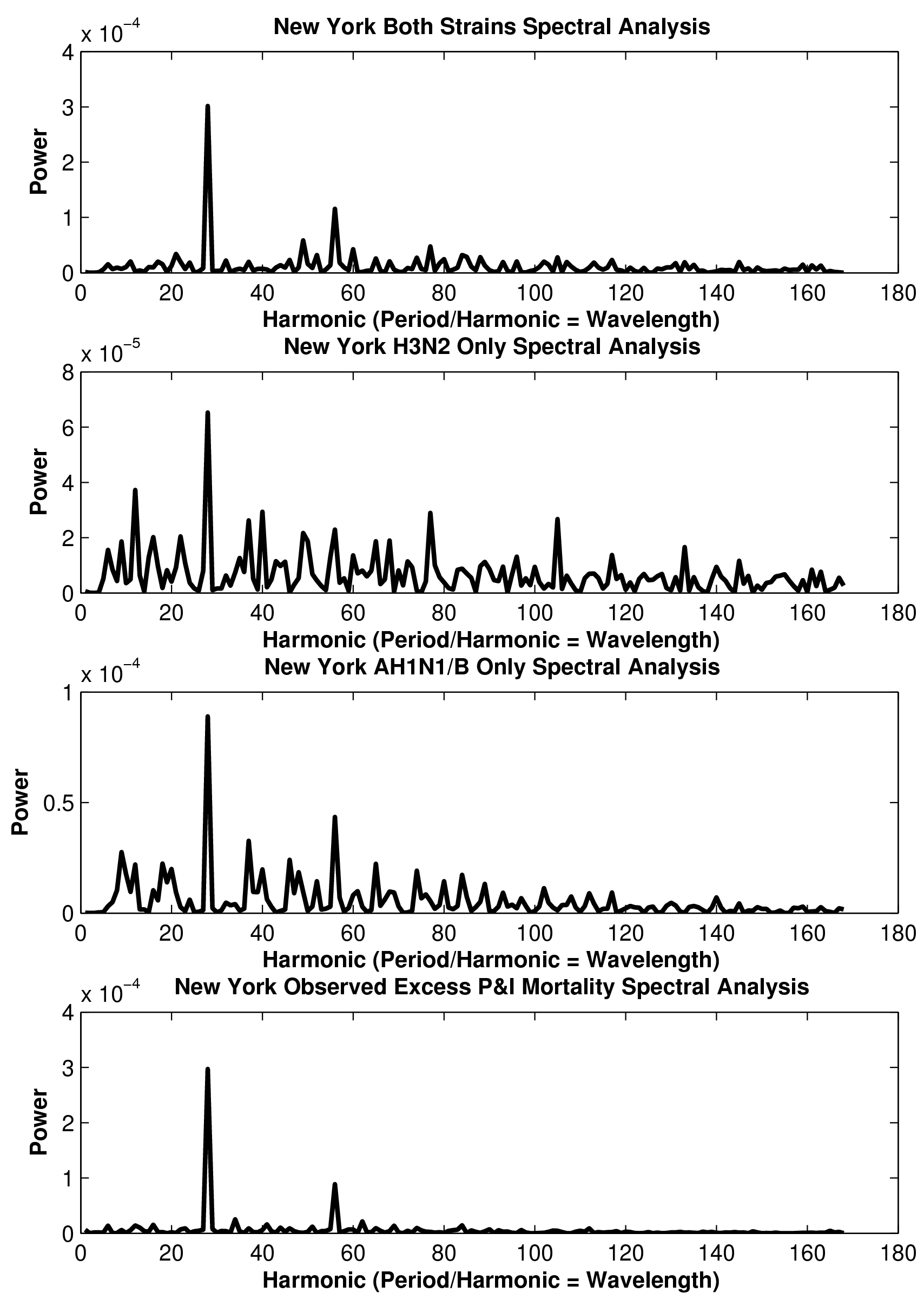

Supplement: Figure S13 — Power spectra of the tenth best-fit New York dual-strain SIRS simulation. Power spectra of a typical (tenth) best-fit New York dual-strain discrete SIRS simulation (top), broken down by subtype (middle two panels), and the New York observed excess P&I mortality data (bottom), 1975–2002. (0.12 MB GIF) [file pbio.1000316.s013.gif]
