# Supplementary material for: Absolute Humidity and the Seasonal Onset of Influenza in the Continental United States
Source: PLoS Biol. 2010 Feb 23;8(2):e1000316. doi: 10.1371/journal.pbio.1000316 (PMC2826374; doi:10.1371/journal.pbio.1000316)
Supplement: Table S1 — Correlation coefficients of daily anomalies in wintertime (October–February) surface meteorological variables for the lower 48 US states and DC across all these sites, 1972–2002. (0.03 MB DOC) [file pbio.1000316.s016.doc]

|  | AH’ | RH’ | Temperature’ | Solar Radiation’ |
| --- | --- | --- | --- | --- |
| AH’ | 1 | 0.12 | 0.89 | 0.20 |
| RH’ | 0.12 | 1 | -0.17 | -0.46 |
| Temperature’ | 0.89 | -0.17 | 1 | 0.34 |
| Solar Radiation’ | 0.20 | -0.46 | 0.34 | 1 |
