# Supplementary material for: Absolute Humidity and the Seasonal Onset of Influenza in the Continental United States
Source: PLoS Biol. 2010 Feb 23;8(2):e1000316. doi: 10.1371/journal.pbio.1000316 (PMC2826374; doi:10.1371/journal.pbio.1000316)
Supplement: Table S2 — Parameter combinations for the ten best-fit dual-strain SIRS simulations at each site with the parameters R 0max , R 0min , D , and L randomly chosen from within specified ranges. Best-fit simulations were selected based on RMS error after scaling the 31-y mean daily infection number to the 31-y mean observed daily excess P&I mortality rate. The scaling factor and correlation with observed mean annual excess P&I mortality rates are also shown. (0.13 MB DOC) [file pbio.1000316.s017.doc]

| State | **Correlation (r)** | **RMS Error** | **L (years)** | **D (days)** |  | $R_{0\text{min}}$ | Scaling Factor  **(x1e-4)** |
| --- | --- | --- | --- | --- | --- | --- | --- |
| Arizona | 0.92 | 0.0059 | 7.52 | 2.68 | 3.81 | 0.87 | 5.32 |
| Arizona | 0.90 | 0.0061 | 8.08 | 2.82 | 4.00 | 0.84 | 5.64 |
| Arizona | 0.92 | 0.0062 | 7.28 | 2.65 | 3.73 | 0.92 | 4.97 |
| Arizona | 0.92 | 0.0062 | 2.28 | 5.64 | 3.17 | 0.84 | 0.29 |
| Arizona | 0.92 | 0.0064 | 8.75 | 2.52 | 3.46 | 1.18 | 7.79 |
| Arizona | 0.92 | 0.0065 | 7.99 | 2.53 | 3.56 | 0.88 | 6.67 |
| Arizona | 0.91 | 0.0065 | 6.53 | 2.14 | 3.76 | 1.01 | 4.98 |
| Arizona | 0.90 | 0.0066 | 9.21 | 2.20 | 3.84 | 0.93 | 9.54 |
| Arizona | 0.91 | 0.0066 | 4.29 | 2.46 | 3.00 | 1.06 | 2.17 |
| Arizona | 0.94 | 0.0067 | 5.55 | 3.13 | 3.85 | 1.01 | 2.44 |
| Florida | 0.97 | 0.0035 | 4.78 | 2.48 | 3.16 | 1.28 | 4.73 |
| Florida | 0.97 | 0.0039 | 5.50 | 2.26 | 2.96 | 1.21 | 7.82 |
| Florida | 0.96 | 0.0043 | 2.75 | 2.80 | 3.33 | 1.12 | 1.60 |
| Florida | 0.96 | 0.0044 | 3.56 | 2.39 | 2.90 | 1.19 | 3.20 |
| Florida | 0.95 | 0.0046 | 2.30 | 2.90 | 3.54 | 1.00 | 1.27 |
| Florida | 0.95 | 0.0046 | 3.02 | 2.40 | 3.43 | 1.23 | 1.97 |
| Florida | 0.94 | 0.0048 | 4.74 | 2.16 | 2.49 | 1.16 | 7.64 |
| Florida | 0.96 | 0.0049 | 2.16 | 4.99 | 3.70 | 1.20 | 0.52 |
| Florida | 0.94 | 0.0049 | 4.89 | 3.16 | 3.80 | 1.27 | 3.67 |
| Florida | 0.95 | 0.0050 | 2.53 | 2.84 | 3.91 | 1.16 | 1.21 |
| Illinois | 0.96 | 0.0031 | 7.85 | 2.41 | 3.72 | 1.27 | 5.88 |
| Illinois | 0.96 | 0.0032 | 3.65 | 2.53 | 2.38 | 1.02 | 1.94 |
| Illinois | 0.96 | 0.0032 | 5.77 | 2.58 | 2.91 | 1.24 | 3.52 |
| Illinois | 0.97 | 0.0032 | 5.61 | 4.00 | 3.61 | 1.27 | 1.92 |
| Illinois | 0.96 | 0.0033 | 5.87 | 2.69 | 3.60 | 1.22 | 3.12 |
| Illinois | 0.96 | 0.0034 | 6.44 | 3.42 | 3.92 | 1.22 | 2.81 |
| Illinois | 0.95 | 0.0034 | 3.24 | 3.27 | 2.85 | 0.96 | 1.01 |
| Illinois | 0.95 | 0.0035 | 9.05 | 2.92 | 2.80 | 1.26 | 8.56 |
| Illinois | 0.95 | 0.0035 | 5.38 | 4.33 | 3.63 | 1.24 | 1.62 |
| Illinois | 0.95 | 0.0036 | 4.06 | 2.41 | 2.58 | 1.16 | 2.14 |
| New York | 0.98 | 0.0035 | 2.76 | 5.89 | 3.91 | 1.27 | 0.45 |
| New York | 0.97 | 0.0036 | 2.58 | 5.25 | 3.56 | 1.16 | 0.47 |
| New York | 0.97 | 0.0036 | 4.29 | 2.46 | 3.00 | 1.06 | 2.88 |
| New York | 0.97 | 0.0037 | 3.31 | 3.67 | 3.95 | 0.94 | 1.01 |
| New York | 0.97 | 0.0037 | 3.39 | 4.39 | 3.86 | 0.87 | 0.93 |
| New York | 0.97 | 0.0037 | 4.20 | 3.27 | 3.96 | 1.21 | 1.72 |
| New York | 0.97 | 0.0037 | 4.49 | 3.11 | 3.18 | 1.01 | 2.41 |
| New York | 0.97 | 0.0038 | 2.49 | 5.32 | 3.65 | 1.04 | 0.43 |
| New York | 0.97 | 0.0038 | 5.44 | 2.18 | 3.62 | 1.06 | 4.67 |
| New York | 0.97 | 0.0039 | 2.88 | 4.13 | 3.29 | 1.13 | 0.76 |
| Washington | 0.94 | 0.0036 | 2.07 | 3.40 | 2.35 | 0.84 | 0.50 |
| Washington | 0.93 | 0.0037 | 4.51 | 3.57 | 3.19 | 0.86 | 1.49 |
| Washington | 0.93 | 0.0037 | 2.16 | 6.86 | 3.61 | 0.81 | 0.18 |
| Washington | 0.93 | 0.0037 | 6.12 | 3.51 | 2.67 | 1.06 | 3.10 |
| Washington | 0.94 | 0.0038 | 6.51 | 4.84 | 3.71 | 0.87 | 2.08 |
| Washington | 0.93 | 0.0038 | 7.28 | 2.65 | 3.73 | 0.92 | 4.44 |
| Washington | 0.93 | 0.0038 | 9.26 | 3.60 | 3.80 | 1.19 | 5.24 |
| Washington | 0.93 | 0.0039 | 7.97 | 3.15 | 3.43 | 1.12 | 4.64 |
| Washington | 0.93 | 0.0039 | 7.90 | 2.03 | 2.39 | 0.86 | 11.04 |
| Washington | 0.93 | 0.0039 | 9.33 | 3.04 | 3.59 | 1.01 | 6.57 |
