# Supplementary material for: Absolute Humidity and the Seasonal Onset of Influenza in the Continental United States
Source: PLoS Biol. 2010 Feb 23;8(2):e1000316. doi: 10.1371/journal.pbio.1000316 (PMC2826374; doi:10.1371/journal.pbio.1000316)
Supplement: Table S3 — Parameter combinations for the ten best-fit simulations at the Arizona, Florida, Illinois New York, and Washington state sites. Five thousand simulations were performed at each site with D = 2.4 d and the three remaining parameters randomly chosen from the ranges: L = 2–8 y, R 0max = 2–4, and R 0min = 1–1.3. Best-fit simulations were selected for the five sites in aggregate based on RMS error after scaling the 31-y mean daily infection number to the 31-y mean observed daily excess P&I mortality rate at each site. The scaling factor itself, representing mortality per infection, is also shown. (0.05 MB DOC) [file pbio.1000316.s018.doc]

| **Rank** | **RMS Error** | **Correlation Coefficient**  **(r)** | **L (years)** | **D (days)** | $R_{0\text{max}}$ | $R_{0\text{min}}$ | Scaling Factor  **(x1e-4)** |
| --- | --- | --- | --- | --- | --- | --- | --- |
| 1 | 0.0071 | 0.85 | 7.06 | 2.40 | 3.60 | 1.23 | 2.08 |
| 2 | 0.0071 | 0.84 | 7.93 | 2.40 | 3.69 | 1.24 | 2.37 |
| 3 | 0.0071 | 0.84 | 7.98 | 2.40 | 3.87 | 1.28 | 2.26 |
| 4 | 0.0071 | 0.84 | 6.53 | 2.40 | 3.18 | 1.26 | 2.10 |
| 5 | 0.0072 | 0.84 | 2.59 | 2.40 | 2.24 | 1.10 | 1.22 |
| 6 | 0.0072 | 0.84 | 4.47 | 2.40 | 2.79 | 1.10 | 1.67 |
| 7 | 0.0072 | 0.84 | 5.72 | 2.40 | 3.41 | 1.17 | 1.77 |
| 8 | 0.0072 | 0.84 | 3.37 | 2.40 | 2.34 | 1.19 | 1.42 |
| 9 | 0.0072 | 0.84 | 6.29 | 2.40 | 3.48 | 1.25 | 1.89 |
| 10 | 0.0072 | 0.84 | 6.62 | 2.40 | 3.53 | 1.12 | 2.03 |
