# Supplementary material for: Absolute Humidity and the Seasonal Onset of Influenza in the Continental United States
Source: PLoS Biol. 2010 Feb 23;8(2):e1000316. doi: 10.1371/journal.pbio.1000316 (PMC2826374; doi:10.1371/journal.pbio.1000316)
Supplement: Table S4 — Comparison of best common-fit model simulation ( Table 1 ) parameter fluctuations at the five sites with those of Dushoff et al., 2004. (0.07 MB DOC) [file pbio.1000316.s019.doc]

|  | AZ  Best Fit | FL  Best Fit | IL  Best Fit | NY  Best Fit | WA  Best Fit | Dushoff et al. |
| --- | --- | --- | --- | --- | --- | --- |
| L (years) | 5.35 | 5.35 | 5.35 | 5.35 | 5.35 | 8.00 |
| D (days) | 3.24 | 3.24 | 3.24 | 3.24 | 3.24 | 9.13 |
| Mean $\beta\left( t \right)$ (number/year) | 243 | 159 | 209 | 223 | 233 | 500 |
| Maximum $\beta\left( t \right)$ (number/year) | 360 | 288 | 386 | 364 | 370 | 502 |
| Minimum $\beta\left( t \right)$ (number/year) | 137 | 130 | 130 | 136 | 149 | 498 |
| Mean $R_{0}\left( t \right)$ (number) | 2.15 | 1.41 | 1.86 | 1.98 | 2.06 | 12.50 |
| Maximum $R_{0}\left( t \right)$ (number) | 3.19 | 2.56 | 3.42 | 3.23 | 3.29 | 12.55 |
| Minimum $R_{0}\left( t \right)$ (number) | 1.21 | 1.16 | 1.16 | 1.21 | 1.32 | 12.45 |
| Mean $T\left( t \right)$ (years) | 1.33 | 2.38 | 1.76 | 1.56 | 1.38 | 0.829 |
| Maximum $T\left( t \right)$ (years) | 2.97 | 3.45 | 3.44 | 3.02 | 2.40 | 0.830 |
| Minimum $T\left( t \right)$ (years) | 0.92 | 1.10 | 0.88 | 0.92 | 0.91 | 0.827 |
