# Supplementary material for: Absolute Humidity and the Seasonal Onset of Influenza in the Continental United States
Source: PLoS Biol. 2010 Feb 23;8(2):e1000316. doi: 10.1371/journal.pbio.1000316 (PMC2826374; doi:10.1371/journal.pbio.1000316)
Supplement: Table S5 — Parameter combinations for the ten best-fit simulations using only the school calendar at the New York state site. Five thousand simulations were performed with the parameters SC, R 0min, D, and L randomly chosen from within specified ranges. Best-fit simulations were selected based on RMS error after scaling the 31-y mean daily infection number to the 31-y mean observed daily excess P&I mortality rate. (0.04 MB DOC) [file pbio.1000316.s020.doc]

| **Rank** | **Correlation Coefficient**  **(r)** | **RMS Error** | **L (years)** | **D (days)** | **SC**  **(unitless)** | $R_{0\text{min}}$ |
| --- | --- | --- | --- | --- | --- | --- |
| 1 | 0.94 | 0.0056 | 7.28 | 2.64 | 1.90 | 0.92 |
| 2 | 0.94 | 0.0057 | 8.81 | 3.47 | 1.88 | 1.05 |
| 3 | 0.94 | 0.0060 | 3.89 | 2.39 | 1.42 | 1.10 |
| 4 | 0.95 | 0.0060 | 4.77 | 3.15 | 1.72 | 1.14 |
| 5 | 0.93 | 0.0060 | 3.46 | 2.63 | 1.64 | 0.99 |
| 6 | 0.94 | 0.0060 | 2.15 | 3.87 | 1.86 | 0.92 |
| 7 | 0.93 | 0.0061 | 7.66 | 2.29 | 1.78 | 1.16 |
| 8 | 0.93 | 0.0063 | 6.89 | 2.39 | 1.44 | 1.12 |
| 9 | 0.93 | 0.0063 | 5.47 | 2.75 | 1.81 | 1.12 |
| 10 | 0.92 | 0.0064 | 8.41 | 3.29 | 1.85 | 1.09 |
