# Supplementary material for: Absolute Humidity and the Seasonal Onset of Influenza in the Continental United States
Source: PLoS Biol. 2010 Feb 23;8(2):e1000316. doi: 10.1371/journal.pbio.1000316 (PMC2826374; doi:10.1371/journal.pbio.1000316)
Supplement: Text S1 — Supporting text providing additional descriptions of methodologies and findings. (0.17 MB DOC) [file pbio.1000316.s021.doc]

**Supplementary Information**

Figure S1 presents weekly time series of pneumonia and influenza (P&I) mortality per 100,000 people in the US, 1972-2002. Mortality peaks each winter season. Excess P&I mortality provides a robust indicator of the timing and impact of influenza epidemics, which measures mortality attributable to influenza above a seasonal baseline.

*Testing the Statistical Significance of* $AH^{'}$ *Prior to Onset*

The statistical significance of the average $AH^{'}$ conditions during the 4 weeks prior to onset was assessed through bootstrapping using a Monte Carlo sampling procedure. The null hypothesis is that $AH^{'}$ conditions preceding epidemic onset do not deviate significantly from the average climatology (i.e. $AH^{'}=0$). Depending on the threshold level used to define epidemic onset, *n* onset events were found for the contiguous 48 states and DC. A maximum of *n* = 1470 events is possible (30 winters x 49 sites), but not all winter-sites exceeded the epidemic onset threshold; for the thresholds used, *n* ranged between 1181 and 1420 events.

To perform the bootstrapping, $AH^{'}$ were sampled randomly *n* times in 4-week continuous blocks beginning from October 1 through February 28, 1972-2002 from all 49 sites. This procedure sampled $AH^{'}$ randomly in both space and time. As the 31-year daily average $AH^{'}$ value from each site is by definition zero, the random sampling across sites is appropriate for the testing our hypothesis. By using continuous 4-week block samples we retain the intrinsic temporal autocorrelation present in local $AH^{'}$time series values. The sampling of *n* 4-week time series was then averaged to provide a synthetic, random-sampled measure of winter season 4-week-block average $AH^{'}$ conditions. This process was repeated 100,000 times to create a synthetic distribution of these randomly sampled averaged values. Statistical significance was assessed on the basis of where the actual value of average $AH^{'}$ conditions during the 4 weeks prior to onset lay on this synthetic distribution. Results of this analysis were not sensitive to the precise duration of time prior to onset; similarly significant results were attained for the period 3 weeks prior to onset.

# *AH and the Regional Onset of Wintertime Influenza Outbreaks*

Figures S2 and S3 show how $AH^{'}$ varies with onset date for various regions of the country. Strongest associations of negative $AH^{'}$ with onset are for the Gulf and Northeast regions. All regions but the Southwest show a tendency for negative $AH^{'}$ during the 3-4 weeks prior to onset. States were grouped into the regions shown in Figure S4 based on gross similarities of climate.

*Association of Other Environmental Variables with Wintertime Influenza Onset*

As detailed in the primary paper, we performed analyses to determine whether other potential environmental drivers of influenza are also associated with the wintertime influenza onset. Onset was defined as before; anomalies were defined as in Equation 1. Anomalous RH, temperature and incident solar radiation conditions were averaged for the period 4 to 0 weeks prior to onset for each of the 30 winters with an influenza outbreak (1972-2002) at each of the contiguous 48 US states and the District of Columbia. All environmental data were derived from NCEP-NCAR reanalysis [23]. Table 1 of the main text shows these values and their bootstrapped significance using a Monte Carlo sampling (100,000 iterations) of each environmental record.

The associations between onset and all 4 environmental variables are statistically significant (excepting solar radiation for the highest onset threshold). The tendencies are for negative anomalies of AH, RH and temperature and positive anomalies of solar insolation during the 4 weeks prior to influenza onset. For AH, RH and temperature, these anomalies are consistent with their expected effects: influenza virus survival and transmission are found to increase at lower AH [11], RH and temperature [14-15,28-30]; however, positive solar insolation anomalies (increased sunshine) are expected to boost vitamin D and melatonin levels [12-13], potentially improving host resistance and reducing influenza transmission. Thus, these results discount a role for solar insolation.

Daily wintertime temperature and AH anomalies are highly collinear (Table S1); daily wintertime AH is also positively correlated with RH and solar insolation levels. However, as described in the main text, certain weather regimes can be associated with negative anomalies of RH, AH and temperature and positive anomalies of solar insolation. In particular, cold northerly air masses, following a cold front, often have cold temperatures, low AH, low RH and cloud-free skies.

*Supplementary SIRS Model Results*

The SIRS model responds to year-to-year differences in AH conditions and simulated susceptibility rates. These differences conspire to create seasonal epidemics with different amplitudes, durations and start dates. Individual outbreak peaks occur throughout the winter (Figure S5). Pronounced double outbreaks (2 winter season peaks) are typically not observed in a simulated individual winter season; however, the average of these individual seasonal outbreaks does produce a dual peak 1972-2002 that matches the average observed 1972-2002 excess P&I mortality (Figure 3) and suggests a clustering of December/January outbreaks and another for February/March.

Figure S6 shows RMS error of the ‘combined fit’ for all 5 state sites (Arizona, Florida, Illinois, New York and Washington) plotted as a function of parameter space for the 5000 parameter combinations used for SIRS modeling. Higher $R_{0\text{max}}$ and low *D* tend to produce lower RMS error. The structure of these plots also shows a region of $R_{0\text{max}}$-*L* parameter space where RMS error is low. In general, higher $R_{0\text{max}}$, higher $R_{0\text{min}}$, lower *L*, and lower *D* favor a better fit with observations.

Table S2 shows the best-fit simulations (based on RMS error) at each of the state sites evaluated individually. As is to be expected, individual site correlations and RMS error for a given parameter combination are better than the combined-fit in which all 5 sites are evaluated simultaneously. Florida, Illinois, New York and Washington yield lower RMS error and higher correlation coefficients than Arizona. All of the sites tend toward high$R_{0\text{max}}$ (>2.8) and low *D* (<4). Best-fit simulations at Florida, Illinois, and New York also tend toward higher $R_{0\text{min}}$ (>1).

Figures S7-S11 show RMS error plotted as function of parameter space for each state. Note that these color scale changes among these figures. As for the combined fit, higher $R_{0\text{max}}$, higher $R_{0\text{min}}$, lower *L*, and lower *D* favor a better fit with observations.

Table S3 shows the results for the 10 best combined fits of the run with *D* = 2.4 days. The improvement to RMS error and correlation coefficients is nominal (compare to Table 2 of the main manuscript). 8 of 10 $R_{0\text{max}}$ > 2.7 (as in Table 2), and $R_{0\text{min}}$ and *L* possess the same range. Similar results were attained for the simulations with *D* = 3.2 days (not shown). The same broad, goodness-of-fit within parameter space is evident, though with *D* fixed, a positive correlation is indicated between $R_{0\text{max}}$and *L*, perhaps reflecting that higher *L* limits population susceptibility due to longer duration of immunity but tis compensated for by higher $R_{0\text{max}}$. Stochasticity within the SIRS model likely is partly responsible for the range of parameter combinations producing simulations that are well-matched to observations.

We also re-ran SIRS model simulations at our 5 main sites (Arizona, Florida, Illinois, New York and Washington) while allowing a 10% chance of cross-protection (i.e. immunity to one strain preventing infection from the other). Best-fit simulation behavior was similar to SIRS simulations with no cross-protection. That is, the model converged to similar parameter space and the quality of fitting based on RMS error and correlation was similar (<3% change) to that presented in Tables 1 and S2.

## Scaling Factor Consistency with Observations

SIRS model simulations produce approximately 10,000-20,000 cases per 100,000 people per year. Estimates of the number of influenza cases in the US range from 15 to 60 million (~5-20% of the population) [5]. Thus, the model infection rate is consistent with infection rate estimates.

For this study we compare model simulated infection rates to observed excess pneumonia and influenza mortality rates. For New York state there were on average a total of 3.6 excess deaths per 100,000 people per year during 1972-2002 (based on observed excess P&I mortality rates). Similar rates are found for Florida and Illinois. Extrapolated to the whole US (300 million people), this is just shy of 11,000 excess deaths due to influenza. Note that while a figure of 36,000 excess deaths is frequently reported, this reflects excess respiratory and circulatory (R&C) deaths, of which pneumonia and influenza deaths, studied here, constitute approximately a 25% subset [31]. Therefore the observed mortality rate per infection is approximately:

[S1]

This value roughly equals the SIRS model scaling factor, which itself represents mortality per infection within the model.

Note that the scaling factor is not an additional SIRS model parameter; rather, it enables, post-simulation, comparison of two disparate data forms: the SIRS model output (infection prevalence) and observed excess P&I mortality rates. The consistency of the scaling factor of best-fit SIRS model simulations with the observed mortality rate per infection (Equation S1) indicates that these best-fit model-simulated epidemics are affecting an appropriate proportion of the model population—i.e. simulated epidemics are neither too large nor too small. The scaling factor itself is calculated for a given location and simulation as follows:

[S2]

where *SF* is the scaling factor, is the mean infectious period in days, $\bar{O}_{c}$ is the 1972-2002 average observed excess P&I mortality rate (per 100,000 people), and $\bar{I}_{c}$ is the 1972-2002 SIRS simulated infection rate (per 100,000 people). For each simulation, the scaling factor is applied prior to calculation of RMS error.

Table 1 in the main text shows the best-fit simulations for the 5 state sites (Arizona, Florida, Illinois, New York state and Washington state) *evaluated together.* That is, it presents the 10 parameter combinations for which the RMS error of simulations averaged over all 5 sites is lowest. For each parameter combination shown in Table 1, the scaling factor is $D_{d}$ times $\bar{O}_{c}$, averaged for all 5 sites, divided by $\bar{I}_{c}$, averaged for all 5 sites; thus, only one scaling factor is estimated.

*Differences in SIRS Model and Real-World Response to Humidity Anomalies*

Analyses of the $AH^{'}$-onset lag relationship in observations (Figure 2) and SIRS model simulations (Figure 4) are largely similar; however, there are some noteworthy differences. The $AH^{'}$-onset lag is about a week shorter in the SIRS model than in observed data. For the observations (Figure 2) pronounced $AH^{'}$ begins 21 days prior to onset as defined by observed excess P&I mortality. In the SIRS model system pronounced$AH^{'}$ appear 15 days prior to onset as defined by model-simulated infection rates (Figure 4). The broader peak of the $AH^{'}$ seen in Figure 2 is likely due to the many other, real-world factors that can mute or accelerate the onset response to $AH^{'}$. This additional, real-world variability likely stems from 2 sources: 1) factors affecting influenza transmission not represented in the SIRS model (e.g. structured interactions of sub-populations within each state that produce mixing and changes in contact among these sub-populations); 2) the spatial structure of the AH and observed excess P&I mortality data within each state that the perfectly-mixed SIRS model does not represent.

Because the SIRS model system does not represent these additional sources of variability, the response to humidity is more immediate, which likely explains the more pronounced $AH^{'}$ prior to onset. Consequently, the SIRS model tends to produce very idealized individual epidemic curves—an exponential rise of infections, an inflection as the infection rate slows, a single peak and then a monotonic fall as the outbreak abates (Figure S5). The observed excess P&I data are less well behaved and exhibit many outbreak forms: single, double and triple peak epidemics, broad plateaus, etc. This difference no doubts reflects the structured interactions of the real world and the broad geography of our sites (entire states) that enable separately-timed outbreaks in distinct communities within a state.

Note, that we have defined onset as the first winter season 2-week period during which simulated daily infection rates exceed a prescribed threshold. The onset date itself is the *last* day of this 2-week period. Given this definition, if negative $AH^{'}$ are indeed facilitating epidemics, these anomalies should appear at least 2-weeks prior to the onset date and continue until onset. This is indeed seen within the model simulations (i.e. Figure 4).

We tested an alternate onset definition that required infection levels exceed prescribed for only one week, and, as expected, this shifted the humidity anomaly to 7 days prior to onset. Similarly, if we define onset as the first day of the 2-week period, all results are shifted 14 days. The *difference* in $AH^{'}$-onset lag between onset defined by SIRS-simulated infection (Figure 4) and onset defined by observed excess P&I mortality (Figure 2) is about 1 week, which roughly corresponds to the median time between infection and mortality.

*Model Simulated Periodicity*

Best-fit SIRS simulations using a single influenza strain (rather than 2) replicated the annual incidence of excess P&I mortality at the state sites very well; however, these simulations possessed power spectra that often exhibited too much power at 2-year and subannual harmonics (Figure S12). These spurious features from the day-to-day output of the 31-year (1972-2002) simulations appear to be due the single strain construct and the fact that the duration of immunity (*L*) ranges from 2-10 years within the SIRS perfectly-mixed population. Longer durations of immunity, and the presence of only a single virus strain, mean that in the years following large outbreaks, much of the population is not susceptible and therefore $R_{E}\left( t \right)<1$. Among the 10 best-fit simulations at each site, there is a negative correlation between the number of outbreaks in each 31-year simulation and *L* (e.g. NY: *r* = -0.40). This association bears out the relationship that with longer mean duration of immunity, there is more time in between outbreaks and a reduced likelihood of annual outbreaks.

Simulations with the dual-strain SIRS, as presented in the main text, did not have this spurious power feature. For these simulations we altered the SIRS model to simulate multiple influenza strains at once with no long-term cross-immunity. Only 2 subtypes were represented: A(H1N1)/B and A(H3N2). Each year beginning in May, the random seeding of infectious individuals in the population (representing emigration/travel) is fixed to the dominant recorded subtype for the U.S. (either A(H1N1)/B or A(H3N2)), based on CDC/MMWR laboratory and antigenic surveillance data.

Figure S13 shows representative power spectra from the 10^th^ best-fit model simulation and observed excess P&I mortality in New York. The individual strains within the dual strain runs behave similarly to the single-strain SIRS model, though they are noisier as they are not always seeded in a given year. The power spectrum of the combined influenza incidence time series (i.e. daily number of cases irrespective of subtype) produces strong peaks at 1 year and 6 months, better matching observations. Similar results are found for the other states.

*Model Endogenous Period and Seasonal Contact Rate Fluctuations*

#

Previous simulations with a similar SIRS model have shown that, given appropriate parameter combinations that yield an endogenous model period of approximately 1 year, a small annual perturbation in the SIRS-model transmission rate, $\beta\left( t \right)$, can produce a dynamical resonance that greatly amplifies the seasonal cycle of infection incidence [32]. It thus could be argued that a small exogenous perturbation could be sufficient to produce the large seasonal changes in influenza incidence observed in temperate regions. However, two findings undermine this argument for dynamical resonance and suggest a more direct, AH-mediated mechanism.

Firstly, the parameter combinations chosen to yield the endogenous period of approximately 1 year in the Dushoff et al. SIRS model [32] required a large basic reproductive number (4≤$R_{0}$≤16). More recent studies indicate that $R_{0}$ for influenza ranges from 1.3 to 3 [16,21,22,24-27]. Our SIRS simulations use parameters that produce a daily $R_{0}\left( t \right)$ consistent with these more recent estimates.

Secondly, the seasonal variability of AH in temperate regions is large (Figure 1d). Average AH conditions during summer in New York state are more than 4 times greater than during winter. Even in Florida summer AH is twice as great as winter. Given these large seasonal changes, and the marked response of influenza virus survival and transmission to these values (Figure 1a and b), the effect of AH on influenza transmission, and therefore $R_{0}$, is great and produces a large annual perturbation in $\beta\left( t \right)$, not a small one (Table S4). Large oscillations may therefore result directly from this large AH-mediated seasonal perturbation.

The SIRS model endogenous period is:

[S3]

where $R_{0}=\beta D$. Dushoff et al. [32] adopted a fixed *D,* varied the contact rate, such that , and used the mean basic reproductive number, $R_{0}=\beta_{0}D$, to calculate *T*. Specifically, As , the annual ranges of $\beta\left( t \right)$, $R_{0}\left( t \right)$, and $T\left( t \right)$ were less than 1% of their respective annual means. Consequently, the endogenous period remained relatively fixed for each simulation.

However, due to the large seasonal fluctuations of AH the endogenous period in our best-fit model simulations changes markedly from winter to summer, and over the course of the entire run ranges from 0.92 to 3.02 years for New York. At all 5 sites, this endogenous period spends much of the year away from the 1-year period needed to produce dynamical resonance in response to an annual forcing (Table S3). Therefore, with a more realistic $R_{0}\left( t \right)$ that varies in response to changing AH, the model endogenous period continuously shifts and resonance is not really possible. Instead, the seasonal fluctuations of influenza infection represent a direct response to the changing reproductive number. When $R_{0}\left( t \right)$ is high, as during winter, the virus flourishes and outbreaks are favored.

# *School Calendar*

Table S5 presents the results of SIRS model simulations for New York state in which $R_{0}\left( t \right)$ was determined by the school calendar. During summer (roughly mid-June through the end of August), , where ; during winter , where $1\leq\text{SC}\leq2$ is the school calendar effect, representing increased IVT due to increased contact among children during the school year. Neither weekends nor school holidays and breaks were observed; that is, $R_{0}\left( t \right)$ remained elevated from the start of the school year until its end. As for the AH simulations, the SIRS model was run for 1972-2002, and model-simulated infection rates were compared with observed excess P&I mortality for New York state.

Best-fit model simulations for New York with the school calendar effect alone do a very good job of capturing the observed seasonal cycle of influenza (Table S5); however, the results do not match the observed excess P&I mortality data as well as with AH alone (Table S2). Using the school calendar RMS error is nearly double, and the model requires a 40-90% change of $R_{0}\left( t \right)$) to simulate seasonality well. Similar results were found for Florida and Illinois. The 40-90% change is larger than the previously estimated modulation of ~25% [19]; however, these previous estimates were derived from a structured population model, so it is difficult to directly compare these findings with ours.

We also ran this same SIRS model but with for weekends and winter and spring holidays during the school year. Best-fit simulations with this structure performed more poorly (RMS error > 0.01; *r* < 0.81 among the 10 best simulations), and failed to capture the observed seasonal cycle of influenza.

Finally, we ran the SIRS model with both the school (no holidays) and AH; however, these results look similar to those of AH alone in terms of quality of simulation. The school calendar effect (*SC*), which acts as a step function increasing $R_{0}\left( t \right)$ for 9 months of each year, essentially rescales $R_{0}\left( t \right)$ during summer, but otherwise parameter combinations are similar. Thus, it is not possible using these model simulations to separate a school term effect from a larger effect of AH on transmissibility.

Figure S14 presents histograms of correlation coefficients for all 5000 simulations of each of the 4 run types performed at New York state: school only (no breaks); school only (with breaks); AH only; school (no breaks) and AH. The AH only simulations produce a distribution of outcomes that possess higher correlation coefficients (mean, minimum and maximum) and lower RMS error (not shown), than school only simulations. The combined school and AH simulation distribution is very similar to that of AH only.

We also re-ran the 10 best New York parameter combinations from the school only (no breaks) (Table S5) and AH only (Table S2) simulations. Each of these parameter combinations was run 100 additional times, each time with different random seeding, to examine the role stochasticity had in producing well-matched simulations (Figure S15). The additional AH only simulations are much more consistently well-matched with observations (mean *r* = 0.912; minimum *r* = 0.670; maximum *r* = 0.981) than the additional school only simulations (mean *r* = 0.704; minimum *r* = -0.024; maximum *r* = 0.962). These findings indicate that the ability of the AH forced simulations to replicate observed excess P&I mortality is less sensitive to stochastic processes within the SIRS model than is the case for the school forced simulations.

**Additional References**

29. Hood AM (1963) Infectivity of influenza virus aerosols. J Hygiene 61: 331-335.

30. Mitchell CA, Guerin LF, Robillard J (1968) Decay of influenza A viruses of human and avian origin. Can J Comp Med 32: 544-546.

31. Thompson WW, et al. (2003) Mortality associated with influenza and respiratory syncytial virus in the United States. JAMA 289: 179-186.

32. Dushoff J, Plotkin JB, Levin SA, Earn DJD (2004) Dynamical resonance can account for seasonality of influenza epidemics. Proc Natl Acad Sci U S A 101: 16915-16916.
